# Large-scale evidence of a general disease (‘*d*’) factor accounting for both mental and physical health disorders in different age groups

**DOI:** 10.1017/S0033291725000522

**Published:** 2025-03-11

**Authors:** Hongyi Sun, Hannah Carr, Miguel Garcia-Argibay, Samuele Cortese, Marco Solmi, Dennis Golm, Valerie Brandt

**Affiliations:** 1 Centre for Innovation in Mental Health, School of Psychology, University of Southampton, Southampton, UK; 2 School of Medical Sciences, Faculty of Medicine and Health, Örebro University, Örebro, Sweden; 3 Department of Medical Epidemiology and Biostatistics, Karolinska Institutet, Stockholm, Sweden; 4 Clinical and Experimental Sciences (CNS and Psychiatry), Faculty of Medicine, University of Southampton, Southampton, UK; 5 Solent NHS Trust, Southampton, UK; 6 Hassenfeld Children’s Hospital at NYU Langone, New York University Child Study Center, New York City, NY, USA; 7 DiMePRe-J-Department of Precision and Rigenerative Medicine-Jonic Area, University of Bari ‘Aldo Moro’, Bari, Italy; 8 SCIENCES Lab, Department of Psychiatry, University of Ottawa, Ontario, Canada; 9 Regional Centre for the Treatment of Eating Disorders and On Track: The Champlain First Episode Psychosis Program, Department of Mental Health, The Ottawa Hospital, Ontario, Canada; 10 Ottawa Hospital Research Institute (OHRI) Clinical Epidemiology Program, University of Ottawa, Ontario, Canada; 11 Department of Child and Adolescent Psychiatry, Charité Universitätsmedizin, Berlin, Germany; 12 Clinic of Psychiatry, Social Psychiatry and Psychotherapy, Hannover Medical School, Hanover, Germany

**Keywords:** Comorbidity, multi-morbidity, “d” factor, lifestyle, wellbeing

## Abstract

**Background:**

It is unknown whether there is a general factor that accounts for the propensity for both physical and mental conditions in different age groups and how it is associated with lifestyle and well-being.

**Methods:**

We analyzed health conditions data from the Millennium Cohort Study (MCS) (age = 17; N = 19,239), the National Child Development Study (NCDS) (age = 44; N = 9293), and the English Longitudinal Study of Ageing (ELSA) (age ≥ 50; N = 7585). The fit of three Confirmatory Factor models was used to select the optimal solution by Comparative Fit Index, Tucker-Lewis Index, and Root Mean Square Error of Approximation. The relationship among *d* factor, lifestyles, and well-being was further explored.

**Results:**

Supporting the existence of the *d* factor, the bi-factor model showed the best model fit in 17-year-olds (MCS:CFI = 0.97, TFI = 0.96, RMSEA = 0.01), 44-year-olds (NCDS:CFI = 0.96, TFI = 0.95, RMSEA = 0.02), and 50+ year-olds (ELSA:CFI = 0.97, TFI = 0.96, RMSEA = 0.02). The *d* factor scores significantly correlated with lifestyle and well-being, suggesting healthier lifestyles were associated with a reduced likelihood of physical and mental health comorbidities, which in turn improved well-being.

**Conclusions:**

Contrary to the traditional dichotomy between mental and physical conditions, our study showed a general factor underlying the comorbidity across mental and physical diseases, related to lifestyle and well-being. Our results inform the conceptualization of mental and physical illness as well as future research assessing risk and pathways of disease transmission, intervention, and prevention. Our results also provide a strong rationale for a systematic screening for mental disorders in individuals with physical conditions and vice versa, and for integrated services addressing multimorbidity.

## Introduction

Health is commonly divided into mental and physical health, although associations between mental and physical disorders are common and present an increasing challenge to researchers and clinical practitioners (Brandt et al., 2023). In the field of mental health, it has been found that symptoms of mental disorders do not cluster in the expected distinct categories that characterize individual mental disorders, such as major depression and generalized anxiety disorder (Caspi et al., 2014). Rather, it has been shown that symptoms of mental illness are all underpinned by a *psychopathology* (‘*p*’) factor that explains the propensity to develop any mental health condition (Caspi et al., 2014). Consistent with this, there has been an increasing body of research focused on transdiagnosticity across mental disorders (Fusar-Poli et al., 2019).

Crucially, comorbidity is common not only among mental conditions but also between mental and physical conditions. Indeed, over the past years, there has been increasing evidence pointing to significant associations between a range of mental and physical conditions. For instance, significant associations have been found between depression and cardiovascular diseases, increased body mass index (BMI), type 2 diabetes, and coronary disease (Hagenaars et al., 2020). Recent meta-analytic evidence in children has confirmed that these associations can be found across a number of mental and physical disorders (Arrondo et al., 2022).

Recently, a wide range of mental and physical conditions in an adult British population was found to be underpinned by a common factor, which was termed the general disease (‘*d*’) factor (Brandt et al., 2023; Cortese et al., 2021), coined to parallel Caspi and colleague’s ‘*p*’ factor. In turn, the term *p* factor was derived from the *g* factor, reflecting a general intelligence dimension that can be broken down into several sub-factors or mental abilities (Caspi et al., 2014). It was proposed that the *p* factor underlies a large number of mental health symptoms, which cluster into specific disorders (Caspi et al., 2014). However, some researchers have voiced criticism about the theoretical and statistical robustness of the *p* factor (Sprooten et al., 2022; Watts et al., 2024). A major concern is that, due to the limitations of the bi-factor model, the models used to define the *p* factor might lead to data overfitting, resulting in high model fit indices that may not correctly reflect the theoretical validity of the constructed models (Dolan & Borsboom, 2023; Watts et al., 2024). Although the *p* factor has now been validated in different populations (Caspi et al., 2014; Sprooten et al., 2022) and similar findings have been verified at the genetic (Sprooten et al., 2022) and neural levels (Xie et al., 2023), different replication studies have shown inconsistency of the *p* factor structure (Eaton et al., 2023), raising concerns about the stability and generalizability across different samples. Furthermore, the correlation and underlying mechanisms between the genomic and neural *p* factor have not been clearly explained (Sprooten et al., 2022), and their existence has not been consistently replicated in different studies (Romer, 2019), questioning the concept of whether *p* factor truly represents a shared vulnerability or is only the result of statistical modeling based on symptom comorbidity (Watts et al., 2024). Overall, the *p* factor is an important concept that has sparked alternative thinking regarding how we view mental health diagnoses and has inspired a multitude of research, partly supporting and partly questioning the concept, as good scientific discourse should.

The *d* factor suggests that having any diagnosis increases the likelihood of receiving other diagnoses, irrespective of whether they are mental or physical in nature. However, important questions about the *d* factor remain unanswered. First, as the *d* factor was tested in one sample only, it is unknown whether the initial findings would be replicated in other samples. Second, it is unclear if the *d* factor is present across different ages, genders, and socio-economic statuses (SES), having been tested only in UK adults. Third, while mental and physical disorders tend to accumulate across the lifespan (Kuan et al., 2019), it is yet unclear why. Several pathways are possible, including genetic propensity, environmental factors, and lifestyle factors. It is also possible that the accumulation of physical disorders increases the risk of developing mental disorders, although the first manifestations of mental disorders commonly already develop in childhood and adolescence.

Unhealthy lifestyle behaviors (e.g. smoking, drinking alcohol, unhealthy diet, and physical inactivity) are commonly considered risk factors for both mental and physical health (Gehlich et al., 2020; Zhang et al., 2021). In order to show the relevance of the *d* factor, we assessed whether lifestyle impacts the *d* factor, and whether the *d* factor is associated with subjective well-being.

Here, we use three large cohorts to empirically test (1) if the *d* factor can be found across different ages; (2) whether lifestyle predicts the *d* factor; and (3) whether the *d* factor is associated with worse later well-being outcomes.

## Methods

### Participants

Three different samples were used in this study. The Millennium Cohort Study (MCS) recruited more than N = 19,000 young people born in the UK between 2000 and 2002 (University of London, 2017). We used an analytic sample of N = 19,239 from the seventh data collection wave in 2018 when cohort members were 17 years old. Additionally, all health data were combined with the previous six waves (Table S1). Both lifestyle factors and well-being outcomes were extracted from the age 17 sweep.

The National Child Development Study (NCDS) recruited N = 17,475 people born in England, Scotland, and Wales in 1958 (Brown & Goodman, 2014). Health conditions were extracted from the biomedical data collection wave in 2002, at age 44. Additionally, medical health data that were not included in the biomedical sweep were taken from sweep 5 (age 33) and sweep 6 (age 42; Table S2). The lifestyle predictors of diet, smoking, and physical activity were extracted from data collection waves at age 33. Alcohol consumption was measured by the alcohol use disorders identification test (AUDIT) (Conigrave et al., 1995) at age 42. The analytic sample comprised N = 9293 adults.

The English Longitudinal Study of Ageing (ELSA) (Banks et al., 2024) recruited a representative UK sample of N = 11,391 participants, aged 50 to 100 years in 2002. We used an analytic sample of N = 7585 from Wave 10 (Table S3), where participants were aged 50 and over (M = 67.91; SD = 9.43) (Banks et al., 2024). Lifestyle factors and well-being outcomes were derived from wave 10 data.

### Measures

#### Health conditions

In the MCS (Table S1), mental health/neurodevelopmental conditions included affective disorders, conduct disorders, ADHD, dyslexia, dyspraxia/dyscalculia, autism spectrum disorder (ASD), and stutter. Physical health conditions included hearing impairments, visual impairments, eczema, asthma, hay fever, food allergy, meningitis, obesity, and epilepsy. Health conditions were self-reported or parent-reported.

In the NCDS (Table S2), mental conditions assessed by the Clinical Interview Schedule Revised (Lewis et al., 1992) included anxiety, phobia, panic disorder, depression, irritability, sleep problems, and forgetfulness/concentration issues. Eating disorders were self-reported through an interview question. Physical health conditions included fatigue, migraine, obesity, heart problems, diabetes, eczema, asthma, hay fever, ulcer, gallstones, IBS, ulcerative colitis/Crohn’s disease, kidney/bladder stones, back pain, arthritis, visual impairments, hearing impairments, tinnitus, and epilepsy. Physical conditions were self-reported or measured at a biomedical sweep.

In the ELSA (Table S3), self-reported mental conditions included depression, anxiety, emotional problems, schizophrenia, psychosis, and bipolar disorder. Self-reported physical health conditions including stroke, hypertension, heart problems, lung disorders, asthma, arthritis, osteoporosis, blood disorders, cancer, Parkinson’s, multiple sclerosis/motor neurone disease, dementia, and diabetes.

#### Lifestyle & well-being

Lifestyle was assessed using four variables in each cohort: alcohol consumption, smoking behavior, diet, and physical activity. Each variable was classified into non-risky behavior (1) and risky behavior (0), according to WHO recommendations (Bull et al., 2020) as follows: smoking was considered risky behavior. Five or more alcoholic drinks per week or an AUDIT drinking score of 20 or above were classified as high risk, a healthy diet with daily intake or 4/5 portions of fruit and/or vegetables per day were considered low risk, more than 1 h of moderate/heavy physical activity per week or regular exercise was considered low risk. The four variables were then added up so that the lifestyle scores range from 0 (high risk) to 4 (low risk).

Well-being was measured using the Warwick Edinburgh Mental Well-Being Scale (WEMWBS; Tennant et al., 2007) in the NCDS and the short version of WEMEBS (Stewart-Brown et al., 2009) in the MCS, with higher scores representing a higher level of mental well-being. The Satisfaction with Life Scale (SWLS; Diener et al., 1985) was used in the ELSA. For consistency in scoring, we reverse-scored the SWLS, with higher scores representing better subjective well-being. Additional information is reported in Table S4.

### Statistical analysis

**Figure 1.**
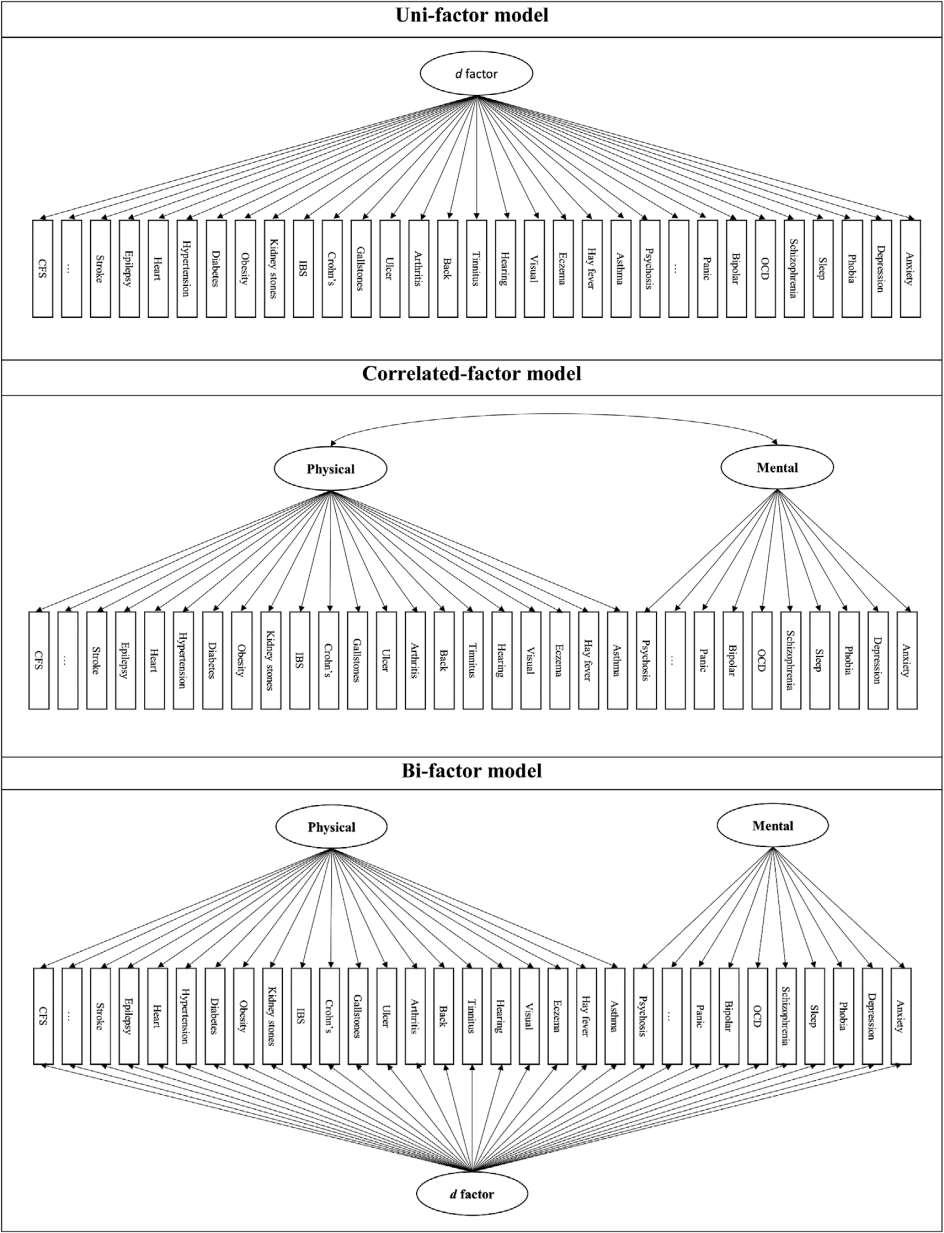
Three confirmatory factor models. *Note*: *d* factor = general disease factor, including all health variables; Mental = mental health factor, including all mental health variables; Physical = physical health factor, including all physical health variables.

Three typical models of Confirmatory Factor Analysis (CFA; Figure 1) were used to compare model fit: (1) a uni-factor model, assuming all health conditions are loadable onto an underlying factor; (2) a correlated factor model, which contains two common factors loaded by health conditions; and (3) a bi-factor model, presuming that health conditions would not just load onto two common factors, but also an underlying dimension. Model fit was assessed by using the Weighted Least Square Mean and Variance adjusted (WLSMV) estimator and compared by Comparative Fit Index (CFI), Tucker–Lewis Index (TLI), and Root Mean Square Error of Approximation (RMSEA). In accordance with current standards, CFI and TLI values >0.95 and RMSEA <0.06 indicated a good model fit (Hu & Bentler, 1999). Then, *d* factor scores were derived from the optimal model and plugged into a mediation model with 95% bootstrapped confidence intervals and 5,000 bootstrap samples to test whether the *d* factor mediates between lifestyle and well-being.

Data analyses were conducted in Mplus v8.3 (Muthén & Muthén, 1998-2011), SPSS v29 (Corp, 2023), and PROCESS v3.5 (Hayes, 2022).

### Measurement invariance and sensitivity analysis

In this study, measurement invariance across gender, ethnicity, and socio-economic status was tested with the suggested process (van de Schoot et al., 2012). However, because all health conditions were categorized in the MCS and ELSA, and were mixed (i.e. categorical and continuous) in the NCDS, only configural and scalar models in the MCS and ELSA were conducted. To further test the robustness and generalizability of findings, the three CFA models were performed in different subgroups (i.e. different genders, ethnicities, and SES) in all three cohorts.

## Results

### Model fit

As reported in Table 1, compared with the uni-factor and correlated factor models, the bi-factor model showed the best model fit in the MCS (CFI = 0.97, TFI = 0.96, RMSEA = 0.01), NCDS (CFI = 0.96, TFI = 0.95, RMSEA = 0.02), and ELSA (CFI = 0.97, TFI = 0.96, RMSEA = 0.02). Supporting the findings from the main analysis, the bi-factor model still showed the best model fit among the subgroup of participants with at least one disease across all three cohorts: MCS (CFI = 0.912, TFI = 0.88, RMSEA = 0.022), NCDS (CFI = 0.907, TFI = 0.89, RMSEA = 0.02), and ELSA (CFI = 0.939, TFI = 0.921, RMSEA = 0.02). The same results have been found in all other sensitivity analyses (Tables S10, S18, and S25).

**Table 1.** Model fit information comparing three models

	Model	Items	Free parameters	Chi^2^ Value	Chi^2^ DF	Chi^2^ p-value	CFI	TFI	RMSEA
MCS	Uni-factor	16	32	4043.39	104	<.001	0.58	0.52	0.04
	Correlated	16	33	1343.76	103	<.001	0.87	0.85	0.03
	Bi-factor	16	48	395.26	88	<.001	0.97	0.96	0.01
NCDS	Uni-factor	28	64	3703.51	350	<.001	0.87	0.86	0.03
	Correlated	28	65	3338.64	349	<.001	0.88	0.87	0.03
	Bi-factor	28	92	1309.19	322	<.001	0.96	0.95	0.02
ELSA	Uni-factor	19	38	1820.72	152	<.001	0.8	0.77	0.04
	Correlated	19	39	729.04	151	<.001	0.93	0.92	0.02
	Bi-factor	19	57	414.86	133	<.001	0.97	0.96	0.02

*Note*: MCS = Millennium Cohort Study; NCDS = 1958 National Child Development Study; ELSA = The English Longitudinal Study of Ageing; Chi^2^ DF = Chi^2^ degree of freedom; CFI = Comparative fit index; TFI = Tucker-Lewis index; RMSEA = Root mean square error of approximation.

### Items loading of bi-factor model

**Figure 2.**
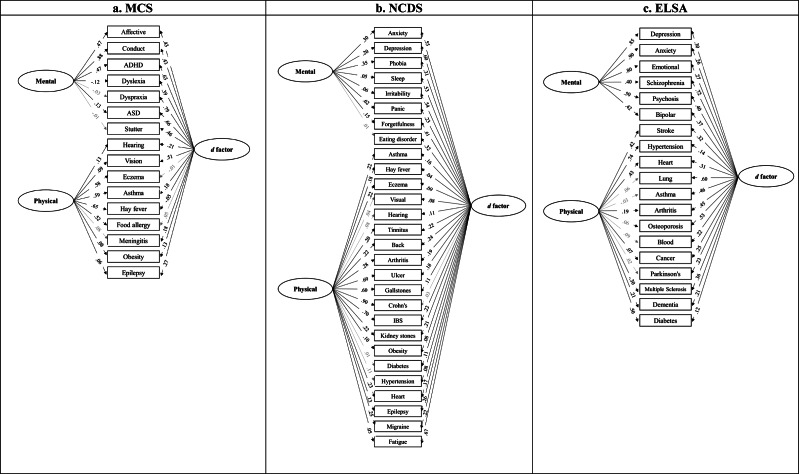
Item loading of bi-factor models. *Note*: (a) Item loading of MCS bi-factor models. (b) Item loading of NCDS bi-factor models. (c) Item loading of ELSA bi-factor models; Grey means non-significant loadings.

In the MCS (Figure 2a), apart from eczema and food allergy not loaded significantly and hay fever loaded negatively on the *d* factor, all other health conditions (13/16) positively and significantly loaded on the *d* factor. The item loading of all mental health conditions and vision impairments was higher than 0.3. More than half of the mental conditions (4/7) loaded onto mental health factors and almost all physical conditions (8/9) loaded onto physical health factors positively and significantly. The item loadings of the MCS subgroup analyses are shown in Tables S11–S17.

In the NCDS (Figure 2b), except for ulcerative colitis/Crohn’s disease, all other health conditions positively and significantly loaded onto the *d* factor. Apart from eating disorders, all other mental conditions loaded on mental health factors positively and significantly. All physical conditions were loaded onto physical health factors, apart from diabetes and hypertension, and hearing and visual impairments. The item loadings of the NCDS subgroup analyses are presented in Tables S19–S24.

In the ELSA (Figure 2c), all health conditions were positively and significantly loaded onto the *d* factor, and the item loading of more than half of the conditions (11/19) was higher than 0.3. All mental conditions positively loaded onto the mental health factor with item loadings above 0.3. Furthermore, more than half of physical conditions (7/13) positively and significantly loaded onto physical health factors and all item loadings of cardio-metabolic conditions (i.e. stroke, hypertension, diabetes, and heart problems) were >0.3. The item loadings of the ELSA subgroup analyses are reported in Tables S26–S32.

### Correlation and Mediation analysis

**Figure 3.**
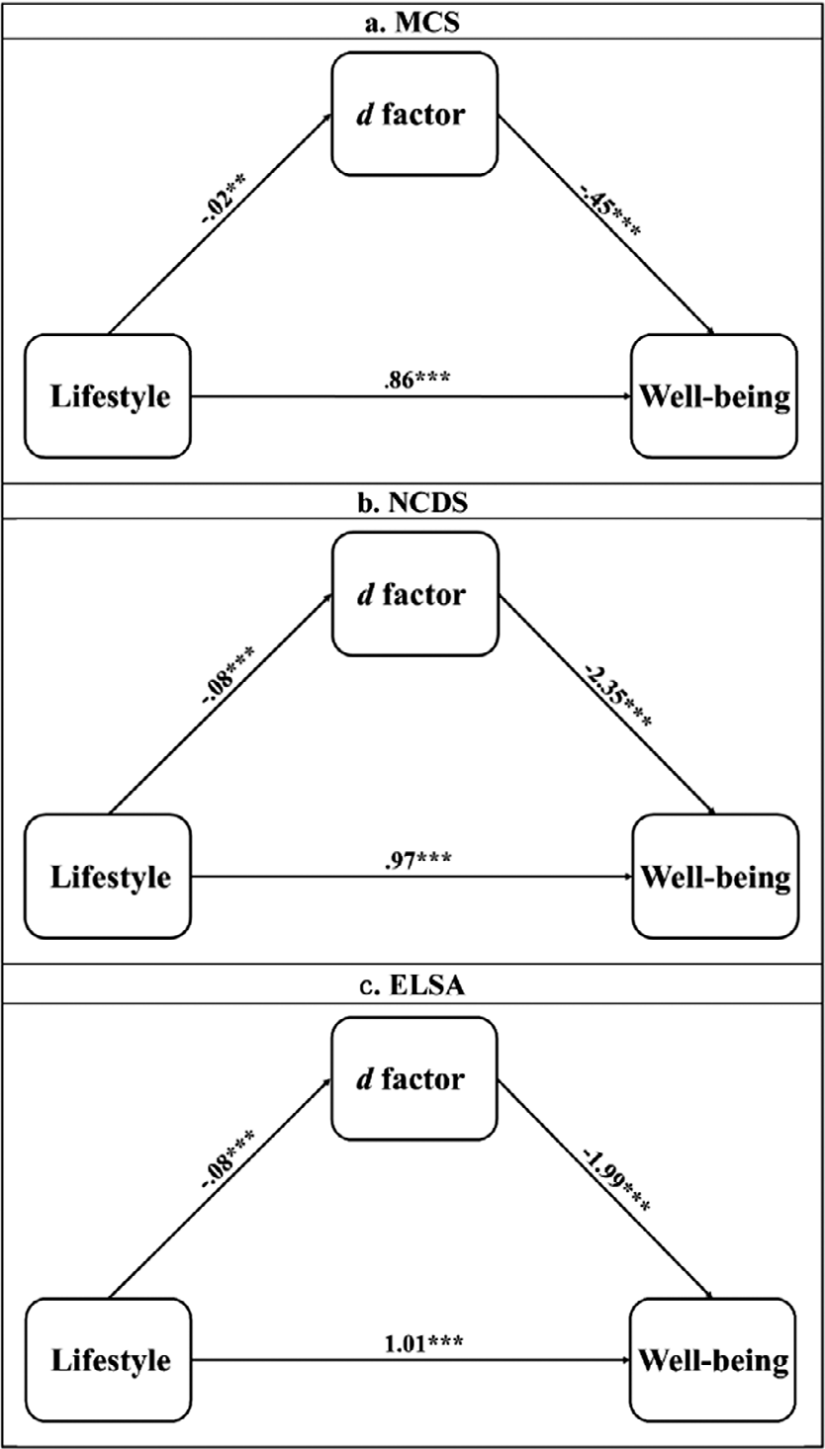
Mediation models. *Note*: (a) Mediation model for MCS; indirect effect: b = .01, 95%CI [.003, .02]. (b) Mediation model for NCDS; indirect effect: b = .19, 95%CI [.14, .25]. (c) Mediation model for ELSA; indirect effect: b = .16, 95% CI [.12, .20]; *p < 0.05, **p < 0.01, ***p < 0.001.

There were significant correlations between lifestyle and the *d* factor score in the MCS (r = −.04, p = .003), NCDS (r = −.10, p < .001), and ELSA (r = −.13, p < .001) studies. Significant correlations were also found between *d*-factor scores and well-being in the MCS (r = −.07, p < .001), NCDS (r = −.27, p < .001), and ELSA (r = −.20, p < .001), suggesting that a healthier lifestyle was associated with a weaker propensity of mental and physical comorbidity, and this was, in turn, associated with higher levels of well-being (Figure 3).

### Measurement invariance

The detailed information on measurement invariance is shown in Table S9. In the MCS, both configural and scalar invariance models showed a sufficient fit, and strong invariance across the gender (ΔCFI = −0.001, ΔRMSEA = −0.001), ethnicity (ΔCFI = 0.002, ΔRMSEA = −0.001), and SES (ΔCFI = 0.002, ΔRMSEA = −0.001) was supported. In the ELSA, configural and scalar invariance models demonstrated an acceptable fit, suggesting acceptable invariance across gender (ΔCFI = −0.011, ΔRMSEA = 0.002) and job status (ΔCFI = −0.013, ΔRMSEA = 0.000).

## Discussion

This is the first study to test the presence of the *d* factor, a factor accounting for the vulnerability to both physical and mental health conditions, across three different samples with ages ranging between 17 and 90+ years. Consistent with a previous ‘*d*’ factor study limited to adults from one sample only (Brandt et al., 2023), we found that the bi-factor model with a common disease or ‘*d*’ factor, a mental health factor, and a physical health factor showed the best model fit in three CFA models in three groups (17, 44, and 50+ years), which suggests that mental and physical health are closely related from a young age. The results of this study expand those from a number of meta-analyses reporting relationships between several specific mental and physical factors in different age groups, for instance between ADHD and a range of physical conditions (Galera et al., 2021), such as asthma (Sun et al., 2021), as well as evidence on the association between a range of mental disorders and heart disease, hypertension, and diabetes (Correll et al., 2017). The results of our study suggest that having any condition increases the likelihood of developing any other condition, mental or physical in nature, providing a unique perspective in understanding comorbidity between mental and physical conditions.

While the *d* factor shows that mental and physical disorders are so closely related that they load on a common factor, it was beyond the scope of this study to investigate why this is the case or which pathways may lead to this association. There are several possible explanations for why mental and physical disorders are closely related from a young age. One of the factors that likely influences physical and mental health is lifestyle. This study indeed showed that a healthier lifestyle predicted lower *d*-factor scores, and those associations increased with age. Moreover, the *d* factor mediated the relationship between lifestyle and well-being. Higher *d*-factor scores were associated with lower well-being with a very small correlation in teenagers and a small to medium correlation in people in their 40s, and a slight decrease in this association above the age of 50. These results extend previous research limited to specific health conditions, which found that better health outcomes are related to a healthier lifestyle including physical activity (Warburton & Bredin, 2017), reduced smoking (Chang et al., 2021), and drinking (Puddephatt et al., 2022; Roerecke & Rehm, 2014), and healthy dietary patterns (Sofi et al., 2014). However, the effects in our study were relatively small, at least when only one point in time was taken into account. Future studies might evaluate long-term exposure to an unhealthy lifestyle.

Although our study did not test further underlying mechanisms, several suggestions can be made based on existing literature. First, it is likely that a range of physical and mental conditions share common genetic polymorphisms that generate a vulnerability towards developing a wide range of diseases. This hypothesis is consistent with studies showing genetic overlap between individual mental conditions and physical conditions. For instance, a recent large Genome Wide Association Study (GWAS) showed that ADHD was genetically correlated with other mental conditions but also with a number of physical factors, such as obesity, diabetes, and arthritis (Demontis et al., 2019). Other associations for which there is evidence include a genetic link between immune abnormalities and mental disorders (Tylee et al., 2018), such as schizophrenia, depression, anorexia nervosa, and Tourette syndrome (Liao et al., 2022). Cardiovascular diseases have also been genetically related to mental disorders (Rodevand et al., 2021), such as schizophrenia, depression, and bipolar disorder. Furthermore, gastrointestinal disorders have been genetically linked with depression (Wu et al., 2021). Additionally, a large study in the Danish genealogy and patient register showed that mental disorders, pulmonary, gastrointestinal, and neurological conditions had similar genetic correlational profiles (Athanasiadis et al., 2022). However, despite high correlations among psychiatric disorders, the concept of a genetic *p* factor has been challenged by some in the field (Grotzinger et al., 2022). For instance, a recent article investigating 11 psychiatric disorders argued that even though all disorders were genetically highly correlated, assuming a single *p* factor would obscure potentially important correlational patterns between genetic background and biobehavioural measures (i.e. accelerometer data) (Grotzinger et al., 2022). Moreover, individual variants (i.e. a mutation in the DNA that can sometimes cause disease) were not well accounted for by a *p* factor (Grotzinger et al., 2022). Thus, future research should test the assumption of a general *d* factor at the genetic level. Additionally, a recent systematic review of 19 meta-analyses indicated a potential trans-diagnostic risk pathway between a risk factor for mental disorders and mortality through common physical diseases (Grummitt et al., 2021). It is also possible that physical ill-health affects mental health, especially in later life. It would be useful to further disentangle how different risk factors and physical health outcomes as well as mental health outcomes are related.

Of note, our findings suggest that physical conditions may also cluster into distinct factors, much like mental conditions do. Mental disorders commonly cluster into internalizing disorders (e.g. depression, anxiety), externalizing disorders (e.g. ADHD, ODD), and thought disorders (e.g. OCD, schizophrenia) (Caspi et al., 2014). In the case of physical conditions included in this study, all cardio-metabolic variables in the ELSA loaded positively onto the physical factor with item loading higher than 0.3, suggesting a possible distinct cluster. Currently, there is no unified theoretical basis on which to cluster physical factors. Future studies might utilize data-driven approaches such as hierarchical clustering or exploratory factor analyses to further explore the factor structure for physical disorders. Additionally, more fine-grained clustering of conditions, beyond differentiating mental and physical health conditions, may also give rise to disorder clusters that challenge our current understanding of human health conditions.

Our results further support current trends in the field of mental health care (Williams et al., 2024) and beyond recognizing that mental and physical symptoms should be diagnosed and treated in a more integrated manner. Even though physical health conditions are often easy to differentiate based on their presentation (e.g. high blood sugar levels may indicate diabetes), certain conditions tend to cluster (e.g. immune conditions and cardio-metabolic conditions) and might not develop independently of each other. Moreover, some conditions are difficult to assign to one domain, such as chronic sleep disorders, pain, and Tourette syndrome. It might therefore be pertinent to develop a more holistic view across mental and physical disorders and test if certain disorders may cluster across these current boundaries. Therefore, even though a dichotomy of mental and physical disorders might be justified and heuristically as well as clinically useful, there is a common underlying dimension that needs to be taken into account in research as well as clinical practice. So far, research has often focused on the mental health consequences of physical disorders, and on exploring risk factors for individual mental or physical disorders. However, our results suggest that mental and physical disorders share a common dimension, possibly related to genetic, socioeconomic, and lifestyle risk factors influencing the vulnerability to develop both mental and physical disorders. Furthermore, our findings stress the need for more comprehensive health screenings that encompass both mental and physical conditions from a young age, and to establish an integrated healthcare system (Solmi et al., 2020).

### Strengths and limitations

The main strengths of our study include using three large and representative UK cohorts to replicate the existence of a *d* factor at different ages. However, the three cohorts used in the study were independent and the results should be replicated in a more generalizable global sample. In addition, most of the health conditions used in the study were self-reported, so the validity of the data may have been affected by biases, including common method variance (Podsakoff et al., 2003), endogeneity (Sande & Ghosh, 2018), recall bias and social desirability/shame.

It would be pertinent to establish how mental and physical comorbidity develops across the lifespan (e.g. childhood), to fully explore potential causal pathways underlying these comorbidities and ensure that findings indicate a distinct *d* factor. Future studies may take into account longitudinal and more comprehensive health measurements (e.g. clinical electronic records), longitudinal lifestyle and environmental factors, as well as genetic factors. One clear limitation of this study is that bi-factor models have been criticized because they tend to have better-fit indices due to their flexibility and may not be the optimal model for comorbidity studies, however, they are particularly useful when the relationship between a general factor and external factors is tested (e.g. the relationship between lifestyle and d; Bornovalova et al., 2020).However, we cannot rule out that the strength of the *d* factor has been overestimated due to the use of a bifactor model. Our current research showed that it was the only model (out of the ones that were tested) that fit the data well across all cohorts and remained stable, irrespective of whether self-ratings or parent-ratings were used.

Another point worth reflecting on is whether the *d* factor could indicate general ‘quality of life’ or ‘well-being’ rather than ‘general disease’ given the inclusion of indicators of internalizing problems (i.e. anxiety and depression). This could provide an alternative explanation for the mediating role of the *d* factor in the association between lifestyle and well-being. Anxiety and depression have, for instance, been found to moderately correlate with physical health conditions such as chronic pain (Dudeney et al., 2024), and could therefore drive the association between physical and mental health problems within the *d* factor. Indeed, the correlation between well-being and the *d* factor is smallest in the MCS cohort, which contains the lowest number of internalizing mental health conditions, and highest in the NCDS cohort which contains mostly internalizing mental health indicators. Even in the NCDS data set, the correlation remains small, making it seem unlikely that the *d* factor merely represents quality of life. Another potential limitation of our mediation analyses is that while mental health and well-being are different constructs, they are correlated. The correlation of depression and anxiety symptoms with well-being is however only moderate showing that well-being as a concept reflects a different experience from internalizing symptoms (Vaingankar et al., 2017).

When examining individual factor loadings onto the *d* factor, neither anxiety nor depression were the highest loading mental health conditions for any of the three cohorts. It should further be considered that the *d* factor comprised of binarized mental health conditions for the MCS and ELSA cohorts which should reduce correlations due to lack of variation within the data. Only, the NCDS cohort included dimensional measures. We however found a significant mediation effect across all three cohorts, with the indirect effect for the NCDS cohort being smaller than the one found for the ELSA cohort. It therefore seems unlikely that the mediation effect is merely caused by an overlap of theoretical constructs (i.e. internalizing problems and well-being).

Future studies may adopt different approaches to further explore related comorbidities. For instance, network modeling has commonly been used in comorbidities studies in recent years (Borsboom, 2017). Unlike traditional latent factor models that explore underlying common dimensions, network models suggest a robust framework for explaining the complex relationship among multiple health conditions (Fried et al., 2017) and define the different comorbidity patterns (Fotouhi et al., 2018). The strength of the network approach lies in its capabilities to capture the dynamic interactions between different health conditions or symptoms, which in turn offers a new view and possibilities for individualized clinical intervention and compensate for the shortcomings of traditional diagnostic classifications (e.g. diagnosis does not correspond to clinical reality; Fried et al., 2017). However, latent models could simplify the structure of complex symptoms or health conditions, particularly in large sample studies (Epskamp et al., 2017). Although we only used latent models to explore the common dimension across mental and physical illnesses in our study, network approaches also provide a valuable perspective and should be further explored in future comorbidity studies. There is also scientific value in considering and testing different models in our attempts to understand and structure complex information.

### Conclusions

A common ‘*d*’ factor may explain an individual’s propensity towards a range of physical and mental conditions across ages. Lifestyle variables, such as poor diet and alcohol intake, are associated with a higher propensity to develop mental and physical conditions and this is associated with lower subjective well-being. Our findings have important implications for research and the organization of healthcare. Ideally, clinicians should consider systematically screening for mental disorders in individuals with physical conditions and vice versa, and health services should be structured to be capable of providing well-integrated care for physical and mental health, which is expected to improve overall outcomes.

## Supporting information

Sun et al. supplementary material Sun et al. supplementary material

## Data Availability

All datasets used for this study are freely available to researchers in the UK via the UK Data Service (https://ukdataservice.ac.uk).

## Supplementary material

The supplementary material for this article can be found at http://doi.org/10.1017/S0033291725000522.
