# Supplementary material for: Large-scale evidence of a general disease (‘d’) factor accounting for both mental and physical health disorders in different age groups
Source: Psychol Med. 2025 Mar 11;55:e78. doi: 10.1017/S0033291725000522 (PMC12080664; doi:10.1017/S0033291725000522)
Supplement: Sun et al. supplementary material [file S0033291725000522sup001.docx]

**Supplemental Online Content**

**Table S1.** Detailed description of the MCS variables.

**Table S2.** Detailed description of the NCDS variables.

**Table S3.** Detailed description of the ELSA variables.

**Table S4.** Detailed description of lifestyle and wellbeing variables.

**Table S5a.** The correlation between *d* factor, lifestyle, and well-being in MCS.

**Table S5b.** The correlation between *d* factor, lifestyle, and well-being in NCDS.

**Table S5c.** The correlation between *d* factor, lifestyle, and well-being in ELSA.

**Table S6.** The detailed item loading of the MCS bi-factor model.

**Table S7.** The detailed item loading of the NCDS bi-factor model.

**Table S8.** The detailed item loading of the ELSA bi-factor model.

**Table S9.** Measurement invariance results.

**Table S10.** Model fit of all MCS subgroups.

**Table S11**. The detailed item loading of the MCS (population with disease) bi-factor model.

**Table S12.** The detailed item loading of the MCS (female) bi-factor model.

**Table S13.** The detailed item loading of the MCS (male) bi-factor model.

**Table S14.** The detailed item loading of the MCS (white population) bi-factor model.

**Table S15.** The detailed item loading of the MCS (non-white population) bi-factor model.

**Table S16.** The detailed item loading of the MCS (high socio-economic status) bi-factor model.

**Table S17.** The detailed item loading of the MCS (low socio-economic status) bi-factor model.

**Table S18.** Model fit of all NCDS subgroups.

**Table S19**. The detailed item loading of the NCDS (population with disease) bi-factor model.

**Table S20.** The detailed item loading of the NCDS (female) bi-factor model.

**Table S21.** The detailed item loading of the NCDS (male) bi-factor model.

**Table S22.** The detailed item loading of the NCDS (white population) bi-factor model.

**Table S23.** The detailed item loading of the NCDS (high socio-economic status) bi-factor model.

**Table S24.** The detailed item loading of the NCDS (low socio-economic status) bi-factor model.

**Table S25.** Model fit of all ELSA subgroups.

**Table S26**. The detailed item loading of the ELSA (population with disease) bi-factor model.

**Table S27.** The detailed item loading of the ELSA (female) bi-factor model.

**Table S28.** The detailed item loading of the ELSA (male) bi-factor model.

**Table S29.** The detailed item loading of the ELSA (white population) bi-factor model.

**Table S30.** The detailed item loading of the ELSA (non-white population) bi-factor model.

**Table S31.** The detailed item loading of the ELSA (with paid job) bi-factor model.

**Table S32.** The detailed item loading of the ELSA (without paid job) bi-factor model.

**Table S1. Detailed description of the MCS variables.**

| **Health condition** | **Yes (%）** | **No (%)** | **Missing (%)** | **Items descriptions** |
| --- | --- | --- | --- | --- |
| ***Mental health condition*** | |  |  |  |
| Affective | 515 (2.6) | 16688 (83.1) | 2879 (14.3) | Strengths and Difficulties Questionnaire (SDQ) [W2-6] |
| Conduct | 1616 (8.0) | 15584 (77.6) | 2882 (14.4) | Strengths and Difficulties Questionnaire (SDQ) [W2-6] |
| ADHD | 1193 (5.9) | 15996 (79.7) | 2893 (14.4) | Strengths and Difficulties Questionnaire (SDQ) [W2-6] |
| Dyslexia | 505 (2.5) | 13051 (65.0) | 6526 (32.5) | Special education reasons/support: Dyslexia  [W2, W6] |
| Dyspraxia/ Dyscalculia | 534 (2.7) | 9718 (48.4) | 9830 (48.9) | Special education reasons/support: Dyspraxia/ Dyscalculia [W2, W6] |
| ASD | 603 (3.0) | 15423 (76.8) | 4056 (20.2) | Whether diagnosed autism [W3-6] |
| Stutter | 409 (2.0) | 15097 (75.2) | 4576 (22.8) | Whether stammer/ stutter [W3, W5, W6] |
| ***Physical health condition*** |  |  |  |  |
| Hearing impairments | 2767 (13.8) | 13477 (67.1) | 5944 (29.6) | Deafness/ partial hearing / hearing problems [W3-7] |
| Visual impairments | 323 (1.6) | 13815 (68.8) | 5944 (29.6) | Blindness/ partial sight/ eyesight problems [W3-7] |
| Eczema | 6915 (34.4) | 9960 (49.6) | 3207 (16.0) | Ever had eczema [W3-6] |
| Asthma | 3439 (17.1) | 12587 (62.7) | 4056 (20.2) | Ever had asthma [W3-6] |
| Hay fever | 5192 (25.9) | 11682 (58.2) | 3208 (16.0) | Ever had hay fever [W3-6] |
| Food allergy | 362 (1.8) | 18877 (94.0) | 843 (4.2) | Allergy to nut, fruit, dairy, wheat, Seafood,  Vegetables, E-numbers [W4] |
| Meningitis | 142 (0.7) | 15251 (75.9) | 4689 (23.3) | Hospital admission: Meningitis [W1, W3, W4] |
| Obesity | 2662 (13.3) | 14520 (72.3) | 2900 (14.4) | Overweight/Obesity Flag [W3-7] |
| Epilepsy | 833 (4.1%) | 14985 (74.6) | 4264 (21.2) | Fits/ Epilepsy/ Seizures [W3-7] |

Note. MCS = Millennium Cohort Study; Affective=affective disorders; ASD= autism spectrum disorder.

**Table S2. Detailed description of the NCDS variables.**

| **Health conditions** | **Yes (%）/Mean (SD)** | **No (%)/ Range** | **Missing (%)** | **Items descriptions** |
| --- | --- | --- | --- | --- |
| ***Mental health condition*** |  |  |  |  |
| Depression | 0.29 (0.73) | 0-4 | 63 (0.7) | Clinical Interview Schedule Revised |
| Anxiety | 0.28 (0.72) | 0-4 | 63 (0.7) | Clinical Interview Schedule Revised |
| Phobia | 0.18 (0.56) | 0-4 | 63 (0.7) | Clinical Interview Schedule Revised |
| Panic disorder | 0.04 (0.35) | 0-4 | 63 (0.7) | Clinical Interview Schedule Revised |
| Irritability | 0.56 (0.92) | 0-4 | 63 (0.7) | Clinical Interview Schedule Revised |
| Sleep problems | 0.74 (1.12) | 0-4 | 63 (0.7) | Any sleep disturbance: Clinical Interview Schedule Revised |
| Forgetfulness | 0.25 (0.70) | 0-4 | 63 (0.7) | Clinical Interview Schedule Revised |
| Eating disorders | 45 (0.5) | 8764 (94.3) | 484 (5.2) | Have you ever had or been told you had an eating problem (Bulimia / Anorexia Nervosa)? Excl. problems with swallowing, some other kind of eating problem [W6] |
| ***Physical health condition*** |  |  |  |  |
| Fatigue | 0.83 (1.34) | 0-4 | 63 (0.7) | Fatigue: Clinical Interview Schedule Revised |
| Migraine | 1829 (19.7) | 7179 (77.3) | 285 (3.1) | Have you ever had or been told you had migraine? [W6] |
| Obesity | 2239 (24.1) | 6906 (74.3) | 148 (1.6) | BMI cut-off; Weight and height measured |
| Heart problems | 146 (1.6) | 8064 (86.8) | 1083 (11.7) | Have you ever suffered from heart trouble? [W5] |
| Hypertension | 84 (0.9) | 9209 (99.1) | 0 | High blood pressure at biomedical sweep |
| Diabetes | 110 (1.2) | 9183 (98.8) | 0 | Have you ever had or been told you had diabetes? [W6] |
| Eczema | 1219 (13.1) | 6903 (74.3) | 1171 (12.6) | Have you ever had or been told you had eczema or psoriasis? [W6] |
| Asthma | 725 (7.8) | 8283 (89.1) | 285 (3.1) | Have you ever had or been told you had asthma? [W6] |
| Hay fever | 1924 (20.7) | 7084 (76.4) | 285 (3.1) | Have you ever had or been told you had hay fever? [W6] |
| Ulcer | 386 (4.2) | 8220 (88.5) | 687 (7.4) | Have you ever had or been told you had an ulcer? [W6] |
| Gallstones | 201 (2.2) | 8300 (89.3) | 792 (8.5) | Have you ever had or been told you had gallstones? [W6] |
| IBS | 784 (8.4) | 7977 (85.8) | 532 (5.7) | Have you ever had or been told you had IBS? [W6] |
| Ulcerative colitis / Crohn’s | 90 (1.0） | 8404 (90.4) | 799 (8.6) | Have you ever had or been told you had ulcerative colitis or Crohn’s disease? [W6] |
| Kidney / bladder stones | 390 (4.2) | 8614 (92.7) | 289 (3.1) | Have you ever had or been told you had kidney / bladder stones [W6]; excl. Nephritis, Infection of kidney/bladder, Blood in urine |
| Back pain | 2875 (30.9) | 6418 (69.1) | 0 | Manchester pain definition: Lower / upper back / spine |
| Arthritis | 1240 (13.3) | 6987 (75.2) | 1066 (11.5) | Have you ever had or been told you had arthritis? [W5] |
| Visual impairments | 537 (5.8) | 8756 (94.2) | 0 | Blind, partially sighted & Vision test: impaired stereo vision, distance vision impaired (>= 6/12) |
| Hearing impairments | 233 (2.5) | 9060 (97.5) | 0 | Have you ever suffered from heart trouble? [W5] |
| Tinnitus | 1272 (13.7) | 7984 (85.9) | 37 (0.4) | Nowadays, do you ever get noises in your head or ears which usually last longer than five minutes at a time, known as tinnitus? |
| Epilepsy | 190 (2.0) | 8814 (94.8) | 289 (3.1) | Have you had or been told you had fits convulsions or epilepsy? [W6] |

Note. NCDS=1958 National Child Development Study; IBS= irritable bowel syndrome; Unlabelled wave-specific items represent those from biomedical sweep.

**Table S3. Detailed description of the ELSA variables.**

| **Health condition** | **Yes (%）** | **No (%)** | **Missing (%)** | **Items descriptions** |
| --- | --- | --- | --- | --- |
| ***Mental health condition*** | |  |  |  |
| Depression | 794 (10.5) | 6788 (89.5) | 4 (0.1) | Psychiatric problem: depression [W10] |
| Anxiety | 710 (9.4) | 6872 (90.6) | 4 (0.1) | Psychiatric problem: anxiety [W10] |
| Emotional | 226 (3.0) | 7356 (97.0) | 4 (0.1) | Psychiatric problem: emotional problems [W10] |
| Schizophrenia | 12 (0.2) | 7570 (99.8) | 4 (0.1) | Psychiatric problem: schizophrenia [W10] |
| Psychosis | 19 (0.3) | 7563 (99.7) | 4 (0.1) | Psychiatric problem: psychosis [W10] |
| Bipolar | 33 (0.4) | 7549 (99.5) | 4 (0.1) | Psychiatric problem: manic depression [W10] |
| ***Physical health condition*** | |  |  |  |
| Stroke | 398 (5.2) | 7186 (94.7) | 2 (<0.1) | Diagnosed stroke [W10] |
| Hypertension | 3300 (43.5) | 1057 (13.9) | 1(<0.1) | Whether ever been told has high blood pressure by doctor [W10] |
| Heart problems | 1057 (13.9) | 6528 (86.1) | 1(<0.1) | Diagnosed congestive heart failure/heart murmur/other heart disease. Excl: abnormal heart rhythm [W10] |
| Lung | 480 (6.3) | 7098 (93.6) | 8 (0.1) | Chronic: diagnosed lung disease [W10] |
| Asthma | 1152 (15.2) | 6428 (84.7) | 6 (0.1) | Chronic: diagnosed asthma [W10] |
| Arthritis | 2995 (39.5) | 4579 (60.4) | 12 (0.2) | Chronic: diagnosed arthritis [W10] |
| Osteoporosis | 643 (8.5) | 6935 (91.4) | 8 (0.1) | Chronic: diagnosed osteoporosis [W10] |
| Blood disorder | 81 (1.1) | 7503 (98.9) | 2 (<0.1) | Chronic: diagnosed blood disorder [W10] |
| Cancer | 1082 (14.3) | 6500 (85.7) | 4 (0.1) | Chronic: diagnosed cancer [W10] |
| Parkinson's | 56 (0.7) | 7526 (99.2) | 4 (0.1) | Chronic: diagnosed Parkinson's disease [W10] |
| Multiple Sclerosis | 34 (0.4) | 7549 (99.5) | 3(<0.1) | Chronic: diagnosed Multiple Sclerosis or Motor Neuron Disease [W10] |
| Dementia | 98 (1.3) | 7485 (98.7) | 3(<0.1) | Chronic: diagnosed dementia/Alzheimer's disease [W10] |
| Diabetes | 953 (12.6) | 6626 (87.3) | 7 (0.1) | Whether confirms diabetes or high blood sugar diagnosis [W10] |

Note. ELSA= The English Longitudinal Study of Ageing; Emotional=emotional disorders.

**Table S4. Detailed description of lifestyle and wellbeing variables.**

|  | **MCS** | **NCDS** | **ELSA** |
| --- | --- | --- | --- |
| **Lifestyle** |  |  |  |
| Smoking | Ever smoked [W7] | Ever smoked [W6] | Ever smoked/ smoking nowadays [W10] |
| Drinking | More than 5 times alcoholic drink in the last 4 weeks [W7] | Alcohol Use Disorders Identification Test (AUDIT) [W6] | More than 5 times alcoholic drink in the last 12 months [W10] |
| Diet | Fruit 2 portions & vegetables 2 portions everyday [W7] | Fresh fruit and raw/cooked vegetables everyday [W6] | 5 portions vegetables/fruit everyday [W10] |
| Exercise | More than 1 day at least an hour of moderate to vigorous physical activity [W7] | Regular exercise [W6] | More than once a week moderate/vigorous sport [W10] |
| Wellbeing | Short version of Warwick Edinburgh Mental Well-Being Scale [W7] | Warwick Edinburgh Mental Well-Being Scale [W8] | Satisfaction with Life Scale [W10] |

Note. MCS = Millennium Cohort Study; NCDS=1958 National Child Development Study; ELSA= The English Longitudinal Study of Ageing.

**Table S5a. The correlation between *d* factor, lifestyle, and well-being in MCS.**

|  | Mean (SD) | d factor | Lifestyle | Well-being |
| --- | --- | --- | --- | --- |
| *d* factor | .12 (.49) |  |  |  |
| Lifestyle | 2.48 (.83) | -.04** |  |  |
| Wellbeing | 22.46 (4.08) | -.07*** | .19*** |  |

Note. MCS = Millennium Cohort Study; *d* factor=general disease factor; *p < 0.05, **p < 0.01, ***p < 0.001.

**Table S5b. The correlation between *d* factor, lifestyle, and well-being in NCDS.**

|  | Mean (SD) | d factor | Lifestyle | Well-being |
| --- | --- | --- | --- | --- |
| *d* factor | .003 (.87) |  |  |  |
| Lifestyle | 2.49 (.89) | -.10*** |  |  |
| Wellbeing | 49.26 (8.12) | -.27** | .13*** |  |

Note. NCDS=1958 National Child Development Study; *d* factor=general disease factor; *p < 0.05, **p < 0.01, ***p < 0.001.

**Table S5c. The correlation between *d* factor, lifestyle, and well-being in ELSA.**

|  | Mean (SD) | d factor | Lifestyle | Well-being |
| --- | --- | --- | --- | --- |
| *d* factor | .09 (.58) |  |  |  |
| Lifestyle | 2.99 (.91) | -.13*** |  |  |
| Wellbeing | 25.17(6.41) | -.20*** | .17*** |  |

Note. ELSA= The English Longitudinal Study of Ageing; *d* factor=general disease factor; *p < 0.05, **p < 0.01, ***p < 0.001.

**Table S6. The detailed item loading of the MCS bi-factor model.**

| **Health condition** | ***d* factor** | **Mental** | **Physical** |
| --- | --- | --- | --- |
| ***Mental health condition*** |  |  |  |
| Affective | 0.430 (0.044) | 0.471 (0.047) |  |
| Conduct | 0.434 (0.050) | 0.875 (0.056) |  |
| ADHD | 0.633 (0.034) | 0.473 (0.046) |  |
| Dyslexia | 0.393 (0.033) | -0.118 (0.051) |  |
| Dyspraxia/ Dyscalculia | 0.793 (0.031) | -0.028 (0.058) |  |
| ASD | 0.657 (0.027) | 0.132 (0.045) |  |
| Stutter | 0.461 (0.035) | -0.006 (0.045) |  |
|  |  |  |  |
| ***Physical health condition*** |  |  |  |
| Hearing impairments | 0.213 (0.022) |  | 0.134 (0.017) |
| Visual impairments | 0.505 (0.037) |  | 0.088 (0.035) |
| Eczema | -0.012 (0.019) |  | 0.579 (0.015) |
| Asthma | 0.181 (0.021) |  | 0.593 (0.016) |
| Hay fever | -0.053 (0.020) |  | 0.654 (0.016) |
| Food allergy | 0.049 (0.041) |  | 0.519 (0.029) |
| Meningitis | 0.178 (0.053) |  | 0.076 (0.046) |
| Obesity | 0.133 (0.021) |  | 0.084 (0.017) |
| Epilepsy | 0.265 (0.029) |  | 0.060 (0.024) |

Note. MCS = Millennium Cohort Study; Affective=affective disorders; Conduct=conduct disorders; ADHD= Attention deficit/ hyperactivity disorder; ASD= autism spectrum disorder; Grey means the results are not significant.

**Table S7. The detailed item loading of the NCDS bi-factor model.**

| **Health condition** | ***d* factor** | **Mental** | **Physical** |
| --- | --- | --- | --- |
| ***Mental health condition*** |  |  |  |
| Depression | 0.598 (0.008) | 0.196 (0.013) |  |
| Anxiety | 0.553 (0.009) | 0.298 (0.014) |  |
| Phobia | 0.309 (0.011) | 0.350 (0.014) |  |
| Panic disorder | 0.231 (0.013) | 0.624 (0.021) |  |
| Irritability | 0.544 (0.009) | 0.057 (0.013) |  |
| Sleep problems | 0.529 (0.010) | 0.048 (0.014) |  |
| Forgetfulness | 0.613 (0.007) | 0.145 (0.012) |  |
| Eating disorders | 0.324 (0.047) | 0.012 (0.033) |  |
|  |  |  |  |
| ***Physical health condition*** |  |  |  |
| Fatigue | 0.671 (0.010) |  | 0.051 (0.014) |
| Migraine | 0.222 (0.016) |  | 0.245 (0.023) |
| Obesity | 0.081 (0.017) |  | 0.095 (0.024) |
| Heart problems | 0.167 (0.036) |  | 0.234 (0.058) |
| Hypertension | 0.083 (0.042) |  | 0.109 (0.071) |
| Diabetes | 0.114 (0.042) |  | 0.008 (0.061) |
| Eczema | 0.091 (0.020) |  | 0.217 (0.028) |
| Asthma | 0.158 (0.021) |  | 0.223 (0.032) |
| Hay fever | 0.038 (0.017) |  | 0.176 (0.024) |
| Ulcer | 0.176 (0.026) |  | 0.687 (0.033) |
| Gallstones | 0.110 (0.031) |  | 0.602 (0.047) |
| IBS | 0.220(0.020) |  | 0.702 (0.024) |
| Ulcerative colitis / Crohn’s | 0.032 (0.046) |  | 0.904 (0.058) |
| Kidney / bladder stones | 0.209 (0.024) |  | 0.219 (0.038) |
| Back pain | 0.241 (0.015) |  | 0.219 (0.021) |
| Arthritis | 0.193 (0.018) |  | 0.276 (0.027) |
| Visual impairments | 0.076 (0.025) |  | 0.039 (0.037) |
| Hearing impairments | 0.114 (0.031) |  | 0.081 (0.047) |
| Tinnitus | 0.221 (0.018) |  | 0.198 (0.026) |
| Epilepsy | 0.256 (0.030) |  | 0.132 (0.050) |

Note. NCDS=1958 National Child Development Study; IBS= irritable bowel syndrome; Grey means the results are not significant.

**Table S8. The detailed item loading of the ELSA bi-factor model.**

| **Health condition** | **d factor** | **Mental** | **Physical** |
| --- | --- | --- | --- |
| ***Mental health condition*** |  |  |  |
| Depression | 0.302 (0.033) | 0.848 (0.020) |  |
| Anxiety | 0.264 (0.034) | 0.899 (0.019) |  |
| Emotional | 0.268 (0.046) | 0.799 (0.023) |  |
| Schizophrenia | 0.722 (0.092) | 0.403 (0.081) |  |
| Psychosis | 0.395 (0.085) | 0.499 (0.090) |  |
| Bipolar | 0.366 (0.081) | 0.419 (0.067) |  |
|  |  |  |  |
| ***Physical health condition*** |  |  |  |
| Stroke | 0.319 (0.052) |  | 0.418 (0.043) |
| Hypertension | 0.142 (0.061) |  | 0.735 (0.043) |
| Heart problems | 0.310 (0.045) |  | 0.425 (0.038) |
| Lung | 0.598 (0.037) |  | 0.055 (0.059) |
| Asthma | 0.457 (0.031) |  | -0.025 (0.046) |
| Arthritis | 0.450 (0.030) |  | 0.192 (0.042) |
| Osteoporosis | 0.525 (0.035) |  | 0.060 (0.053) |
| Blood disorder | 0.224 (0.067) |  | 0.093 (0.069) |
| Cancer | 0.246 (0.031) |  | 0.071 (0.034) |
| Parkinson's | 0.234 (0.052) |  | 0.023 (0.081) |
| Multiple Sclerosis | 0.357 (0.056) |  | -0.302 (0.107) |
| Dementia | 0.214 (0.046) |  | 0.211 (0.061) |
| Diabetes | 0.123 (0.050) |  | 0.501 (0.035) |

Note. ELSA= The English Longitudinal Study of Ageing; Emotional=emotional disorders; Grey means the results are not significant.

**Table S9a. Measurement invariance results for ethnicity for the MCS cohort.**

|  | **χ²** | ***df*** | **p** | **CFI** | **TLI** | **SRMR** | **RMSEA** | **ΔCFI** | **ΔRMSEA** |
| --- | --- | --- | --- | --- | --- | --- | --- | --- | --- |
| **Configural model** | 439.429*** | 176 | <.001 | 0.966 | 0.953 | 0.05 | 0.013 |  |  |
| **Scalar model** | 446.343*** | 202 | <.001 | 0.968 | 0.962 | 0.054 | 0.012 | 0.002 | -0.001 |

**Table S9b. Measurement invariance results for socio-economic status for the MCS cohort.**

|  | **χ²** | ***df*** | **p** | **CFI** | **TLI** | **SRMR** | **RMSEA** | **ΔCFI** | **ΔRMSEA** |
| --- | --- | --- | --- | --- | --- | --- | --- | --- | --- |
| **Configural model** | 441.272*** | 176 | <.001 | 0.966 | 0.954 | 0.054 | 0.013 |  |  |
| **Scalar model** | 454.977*** | 202 | <.001 | 0.968 | 0.962 | 0.059 | 0.012 | 0.002 | -0.001 |

**Table S9c. Measurement invariance results for gender for the MCS cohort.**

|  | **χ²** | ***df*** | **p** | **CFI** | **TLI** | **SRMR** | **RMSEA** | **ΔCFI** | **ΔRMSEA** |
| --- | --- | --- | --- | --- | --- | --- | --- | --- | --- |
| **Configural model** | 444.535*** | 176 | <.001 | 0.968 | 0.956 | 0.058 | 0.013 |  |  |
| **Scalar model** | 478.030*** | 202 | <.001 | 0.967 | 0.96 | 0.063 | 0.012 | -0.001 | -0.001 |

**Table S9d. Measurement invariance results for job state for the ELSA cohort.**

|  | **χ²** | ***df*** | **p** | **CFI** | **TLI** | **SRMR** | **RMSEA** | **ΔCFI** | **ΔRMSEA** |
| --- | --- | --- | --- | --- | --- | --- | --- | --- | --- |
| Configural model | 785.769*** | 234 | <.001 | 0.922 | 0.898 | 0.102 | 0.025 |  |  |
| Scalar model | 901.798*** | 264 | <.001 | 0.909 | 0.895 | 0.115 | 0.025 | -0.013 | 0.000 |

**Table S9e. Measurement invariance results for gender for the ELSA cohort.**

|  | **χ²** | ***df*** | **p** | **CFI** | **TLI** | **SRMR** | **RMSEA** | **ΔCFI** | **ΔRMSEA** |
| --- | --- | --- | --- | --- | --- | --- | --- | --- | --- |
| **Configural model** | 631.543*** | 234 | <.001 | 0.946 | 0.929 | 0.102 | 0.021 |  |  |
| **Scalar model** | 782.263*** | 264 | <.001 | 0.929 | 0.918 | 0.101 | 0.023 | -0.011 | 0.002 |

Note. *df* = degrees of freedom; CFI = comparative fit index; TLI = Tucker–Lewis index; SRMR = standardized root mean square residual; RMSEA = root mean square error of approximation; CI = confidence interval; ^***^*p*<.001; Metric model can not be tested due to binary variables with the WLSMV estimator.

**Table S10. Model fit of all MCS subgroups.**

|  | **Model** | **Items** | **Free parameters** | **Chi^2^ Value** | **Chi^2^ DF** | **Chi^2^ p-value** | **CFI** | **TFI** | **RMSEA** |
| --- | --- | --- | --- | --- | --- | --- | --- | --- | --- |
| **Diseased**  **(N=13179)** | Uni-factor | 16 | 32 | 1370.069 | 104 | <.001 | 0.797 | 0.765 | 0.03 |
|  | Correlated | 16 | 33 | 1100.793 | 103 | <.001 | 0.84 | 0.813 | 0.027 |
|  | Bi-factor | 16 | 48 | 635.713 | 88 | <.001 | 0.912 | 0.88 | 0.022 |
|  |  |  |  |  |  |  |  |  |  |
| **Female**  **(N=8777)** | Uni-factor | 16 | 32 | 1747.857 | 104 | <.001 | 0.531 | 0.459 | 0.042 |
|  | Correlated | 16 | 33 | 569.926 | 103 | <.001 | 0.867 | 0.845 | 0.023 |
|  | Bi-factor | 16 | 48 | 238.403 | 88 | <.001 | 0.957 | 0.941 | 0.014 |
|  |  |  |  |  |  |  |  |  |  |
| **Male**  **(N=9206)** | Uni-factor | 16 | 32 | 2050.684 | 104 | <.001 | 0.596 | 0.533 | 0.045 |
|  | Correlated | 16 | 33 | 727.045 | 103 | <.001 | 0.87 | 0.849 | 0.026 |
|  | Bi-factor | 16 | 48 | 204.674 | 88 | <.001 | 0.976 | 0.967 | 0.012 |
|  |  |  |  |  |  |  |  |  |  |
| **White**  **（N=14815）** | Uni-factor | 16 | 32 | 3005.697 | 104 | <.001 | 0.6 | 0.538 | 0.043 |
|  | Correlated | 16 | 33 | 1061.827 | 103 | <.001 | 0.868 | 0.846 | 0.025 |
|  | Bi-factor | 16 | 48 | 346.434 | 88 | <.001 | 0.964 | 0.951 | 0.014 |
|  |  |  |  |  |  |  |  |  |  |
| **Non-White**  **（N=3121）** | Uni-factor | 16 | 32 | 749.238 | 104 | <.001 | 0.493 | 0.415 | 0.045 |
|  | Correlated | 16 | 33 | 243.598 | 103 | <.001 | 0.889 | 0.871 | 0.021 |
|  | Bi-factor | 16 | 48 | 110.257 | 88 | <.001 | 0.982 | 0.976 | 0.009 |
|  |  |  |  |  |  |  |  |  |  |
| **High SES**  **(N=11293)** | Uni-factor | 16 | 32 | 2144.686 | 104 | <.001 | 0.537 | 0.465 | 0.042 |
|  | Correlated | 16 | 33 | 744.208 | 103 | <.001 | 0.854 | 0.83 | 0.023 |
|  | Bi-factor | 16 | 48 | 259.761 | 88 | <.001 | 0.961 | 0.947 | 0.013 |
|  |  |  |  |  |  |  |  |  |  |
| **Low SES**  **(N=6596)** | Uni-factor | 16 | 32 | 1315.712 | 104 | <.001 | 0.654 | 0.601 | 0.042 |
|  | Correlated | 16 | 33 | 482.007 | 103 | <.001 | 0.892 | 0.874 | 0.024 |
|  | Bi-factor | 16 | 48 | 181.156 | 88 | <.001 | 0.973 | 0.964 | 0.013 |

Note. Chi^2^ DF = Chi^2^ degree of freedom; CFI = Comparative fit index; TFI = Tucker-Lewis index; RMSEA = Root mean square error of approximation.

**Table S11. The detailed item loading of the MCS (population with disease) bi-factor model.**

| **Health condition** | D factor loading | Mental | Physical |
| --- | --- | --- | --- |
| ***Mental health condition*** |  |  |  |
| Affective | 0.585 (0.023) | -0.035(0.057) |  |
| Conduct | 0.943 (0.031) | -0.330 (0.100) |  |
| ADHD | 0.752 (0.019) | 0.165 (0.059) |  |
| Dyslexia | 0.183 (0.038) | 0.323 (0.048) |  |
| Dyspraxia/ Dyscalculia | 0.500 (0.046) | 0.582 (0.060) |  |
| ASD | 0.474 (0.035) | 0.407 (0.051) |  |
| Stutter | 0.261 (0.037) | 0.327 (0.048) |  |
|  |  |  |  |
| ***Physical health condition*** |  |  |  |
| Hearing impairments | 0.058 (0.020) |  | -0.121 (0.025) |
| Visual impairments | 0.358 (0.036) |  | 0.040 (0.052) |
| Eczema | -0.279 (0.017) |  | 0.347 (0.022) |
| Asthma | -0.006 (0.020) |  | 0.414 (0.026) |
| Hay fever | -0.268 (0.018) |  | 0.421 (0.024) |
| Food Allergy | -0.086 (0.025) |  | 0.501 (0.046) |
| Meningitis | 0.117 (0.049) |  | -0.032 (0.062) |
| Obesity | -0.026 (0.019) |  | -0.269 (0.028) |
| Epilepsy | 0.154 (0.028) |  | -0.086 (0.036) |

Note. Grey means the results are not significant.

**Table S12. The detailed item loading of the MCS (female) bi-factor model.**

| **Health condition** | D factor loading | Mental | Physical |
| --- | --- | --- | --- |
| ***Mental health condition*** |  |  |  |
| Affective | 0.476 (0.068) | 0.462 (0.072) |  |
| Conduct | 0.491 (0.077) | 0.755 (0.081) |  |
| ADHD | 0.664 (0063) | 0.519(0.080) |  |
| Dyslexia | 0.346 (0.056) | -0.089 (0.093) |  |
| Dyspraxia/ Dyscalculia | 0.875 (0.067) | -0.108 (0.128) |  |
| ASD | 0.488 (0.060) | 0.168 (0.085) |  |
| Stutter | 0.431 (0.071) | -0.017 (0.094) |  |
|  |  |  |  |
| ***Physical health condition*** |  |  |  |
| Hearing impairments | 0.210 (0.035) |  | 0.197 (0.026) |
| Visual impairments | 0.556 (0.054) |  | 0.148 (0.053) |
| Eczema | -0.079 (0.030) |  | 0.572 (0.023) |
| Asthma | 0.125 (0.036) |  | 0.602 (0.025) |
| Hayfever | -0.099 (0.034) |  | 0.630 (0.024) |
| FoodAllergy | -0.102 (0.067) |  | 0.554 (0.046) |
| Meningitis | 0.247 (0.074) |  | -0.008 (0.072) |
| Obesity | 0.095 (0.033) |  | 0.132 (0.026) |
| Epilepsy | 0.253 (0.048) |  | 0.070 (0.036) |

Note. Grey means the results are not significant.

**Table S13. The detailed item loading of the MCS (male) bi-factor model.**

| **Health condition** | D factor loading | Mental | Physical |
| --- | --- | --- | --- |
| ***Mental health condition*** |  |  |  |
| Affective | 0.474 (0.056) | 0.447 (0.069) |  |
| Conduct | 0.534 (0.067) | 0.834 (0.085) |  |
| ADHD | 0.654 (0.041) | 0.394 (0.063) |  |
| Dyslexia | 0.389 (0043) | -0.169 (0.068) |  |
| Dyspraxia/ Dyscalculia | 0.751 (0.040) | -0.111 (0.077) |  |
| ASD | 0.699 (0.032) | 0.032 (0.063) |  |
| Stutter | 0.438 (0.044) | -0.085 (0.060) |  |
|  |  |  |  |
| ***Physical health condition*** |  |  |  |
| Hearing impairments | 0.185 (0.029) |  | 0.116 (0.024) |
| Visual impairments | 0.508 (0.051) |  | 0.129 (0.050) |
| Eczema | -0.007 (0.025) |  | 0.574 (0.022) |
| Asthma | 0.179 (0.028) |  | 0.593 (0.022) |
| Hayfever | -0.082 (0.026) |  | 0.674 (0.024) |
| FoodAllergy | 0.061 (0.054) |  | 0.505 (0.039) |
| Meningitis | 0.185 (0.084) |  | 0.171 (0.059) |
| Obesity | 0.134 (0.028) |  | 0.068 (0.025) |
| Epilepsy | 0.218 (0.039) |  | 0.072 (0.035) |

Note. Grey means the results are not significant.

**Table S14. The detailed item loading of the MCS (white population) bi-factor model.**

| **Health condition** | D factor loading | Mental | Physical |
| --- | --- | --- | --- |
| ***Mental health condition*** |  |  |  |
| Affective | 0.53 (0.047) | 0.400 (0.059) |  |
| Conduct | 0.546 (0.057) | 0.725 (0.067) |  |
| ADHD | 0.697 (0.040) | 0.439 (0.060) |  |
| Dyslexia | 0.371 (0.036) | -0.157 (0.061) |  |
| Dyspraxia/ Dyscalculia | 0.827 (0.038) | -0.228 (0.084) |  |
| ASD | 0.666 (0.027) | 0.075 (0.058) |  |
| Stutter | 0.447 (0.039) | -0.081 (0.058) |  |
|  |  |  |  |
| ***Physical health condition*** |  |  |  |
| Hearing impairments | 0.181 (0.024) |  | 0.147 (0.019) |
| Visual impairments | 0.478 (0.040) |  | 0.090 (0.040) |
| Eczema | -0.019 (0.021) |  | 0.556 (0.017) |
| Asthma | 0.183 (0.023) |  | 0.581 (0.018) |
| Hayfever | -0.070 (0.022) |  | 0.678 (0.020) |
| FoodAllergy | 0.035 (0.045) |  | 0.478 (0.034) |
| Meningitis | 0.176 (0.056) |  | 0.064 (0.049) |
| Obesity | 0.116 (0.023) |  | 0.101 (0.020) |
| Epilepsy | 0.227 (0.032) |  | 0.072 (0.027) |

Note. Grey means the results are not significant.

**Table S15. The detailed item loading of the MCS (non-white population) bi-factor model.**

| **Health condition** | D factor loading | Mental | Physical |
| --- | --- | --- | --- |
| ***Mental health condition*** |  |  |  |
| Affective | 0.175 (0.100) | 0.645 (0.074) |  |
| Conduct | 0.260 (0.089) | 0.953 (0.085) |  |
| ADHD | 0.434 (0.082) | 0.565 (0.066) |  |
| Dyslexia | 0.434 (0.110) | 0.084 (0.111) |  |
| Dyspraxia/ Dyscalculia | 0.609 (0.095) | 0.366 (0.097) |  |
| ASD | 0.483 (0.091) | 0.166 (0.089) |  |
| Stutter | 0.502 (0.093) | 0.094 (0.086) |  |
|  |  |  |  |
| ***Physical health condition*** |  |  |  |
| Hearing impairments | 0.378 (0.059) |  | 0.147 (0.051) |
| Visual impairments | 0.796 (0.091) |  | 0.188 (0.089) |
| Eczema | -0.058 (0.061) |  | 0.676 (0.036) |
| Asthma | 0.188 (0.064) |  | 0.636 (0.037) |
| Hayfever | -0.033 (0.059) |  | 0.629 (0.035) |
| FoodAllergy | -0.091 (0.108) |  | 0.728 (0.059) |
| Meningitis | 0.445 (0.591) |  | 0.259 (0.120) |
| Obesity | 0.196 (0.057) |  | 0.043 (0.040) |
| Epilepsy | 0.240 (0.090) |  | 0.029 (0.065) |

Note. Grey means the results are not significant.

**Table S16. The detailed item loading of the MCS (high socio-economic status) bi-factor model.**

| **Health condition** | D factor loading | Mental | Physical |
| --- | --- | --- | --- |
| ***Mental health condition*** |  |  |  |
| Affective | 0.423 (0.071) | 0.452 (0.082) |  |
| Conduct | 0.455 (0.072) | 0.761 (0.090) |  |
| ADHD | 0.683 (0.049) | 0.449 (0.074) |  |
| Dyslexia | 0.412 (0.042) | -0.118 (0.074) |  |
| Dyspraxia/ Dyscalculia | 0.835 (0.044) | -0.198 (0.101) |  |
| ASD | 0.684 (0.034) | 0.159 (0.069) |  |
| Stutter | 0.460 (0.046) | -0.063 (0.072) |  |
|  |  |  |  |
| ***Physical health condition*** |  |  |  |
| Hearing impairments | 0.186 (0.029) |  | 0.113 (0.022) |
| Visual impairments | 0.531 (0.049) |  | 0.082 (0.048) |
| Eczema | 0.000 (0.025) |  | 0.571 (0.019) |
| Asthma | 0.176 (0.029) |  | 0.606 (0.021) |
| Hayfever | -0.040 (0.027) |  | 0.681 (0.021) |
| FoodAllergy | 0.055 (0.053) |  | 0.520 (0.035) |
| Meningitis | 0.142 (0.075) |  | 0.096 (0.064) |
| Obesity | 0.134 (0.028) |  | 0.071 (0.023) |
| Epilepsy | 0.278 (0.039) |  | 0.067 (0.031) |

Note. Grey means the results are not significant.

**Table S17. The detailed item loading of the MCS (low socio-economic status) bi-factor model.**

| **Health condition** | D factor loading | Mental | Physical |
| --- | --- | --- | --- |
| ***Mental health condition*** |  |  |  |
| Affective | 0.416 (0.067) | 0.464 (0.068) |  |
| Conduct | 0.425 (0.080) | 0.899 (0.083) |  |
| ADHD | 0.591 (0.057) | 0.493 (0.070) |  |
| Dyslexia | 0.358 (0.062) | -0.087 (0.080) |  |
| Dyspraxia/ Dyscalculia | 0.744 (0.057) | 0.055 (0.085) |  |
| ASD | 0.618 (0.051) | 0.108 (0.071) |  |
| Stutter | 0.454 (0.062) | -0.026 (0.072) |  |
|  |  |  |  |
| ***Physical health condition*** |  |  |  |
| Hearing impairments | 0.255 (0.038) |  | 0.198 (0.031) |
| Visual impairments | 0.492 (0.061) |  | 0.141 (0.055) |
| Eczema | 0.013 (0.033) |  | 0.579 (0.027) |
| Asthma | 0.171 (0.036) |  | 0.585 (0.028) |
| Hayfever | -0.078 (0.035) |  | 0.622 (0.029) |
| FoodAllergy | 0.022 (0.077) |  | 0.543 (0.058) |
| Meningitis | 0.247 (0.093) |  | 0.012 (0.066) |
| Obesity | 0.059 (0.035) |  | 0.139 (0.029) |
| Epilepsy | 0.179 (0.052) |  | 0.044 (0.43) |

Note. Grey means the results are not significant.

**Table S18. Model fit of all NCDS subgroups.**

|  | **Model** | **Items** | **Free parameters** | **Chi^2^ Value** | **Chi^2^ DF** | **Chi^2^ p-value** | **CFI** | **TFI** | **RMSEA** |
| --- | --- | --- | --- | --- | --- | --- | --- | --- | --- |
| Diseased  (N=4310) | Uni-factor | 28 | 64 | 2156.927 | 350 | <.001 | 0.693 | 0.669 | 0.035 |
|  | Correlated | 28 | 65 | 1564.536 | 349 | <.001 | 0.794 | 0.776 | 0.028 |
|  | Bi-factor | 28 | 92 | 872.21 | 322 | <.001 | 0.907 | 0.89 | 0.02 |
|  |  |  |  |  |  |  |  |  |  |
| Female  (N=4681) | Uni-factor | 28 | 64 | 1873.817 | 350 | <.001 | 0.875 | 0.865 | 0.03 |
|  | Correlated | 28 | 65 | 1758.954 | 349 | <.001 | 0.884 | 0.874 | 0.029 |
|  | Bi-factor | 28 | 92 | 788.79 | 322 | <.001 | 0.962 | 0.955 | 0.018 |
|  |  |  |  |  |  |  |  |  |  |
| Male  (N=4612) | Uni-factor | 28 | 64 | 1903.316 | 350 | <.001 | 0.86 | 0.849 | 0.031 |
|  | Correlated | 28 | 65 | 1657.439 | 349 | <.001 | 0.882 | 0.872 | 0.029 |
|  | Bi-factor | 28 | 92 | 762.644 | 322 | <.001 | 0.96 | 0.953 | 0.017 |
|  |  |  |  |  |  |  |  |  |  |
| White  (N=8831) | Uni-factor | 28 | 64 | 3491.353 | 350 | <.001 | 0.867 | 0.857 | 0.032 |
|  | Correlated | 28 | 65 | 3144.147 | 349 | <.001 | 0.882 | 0.872 | 0.03 |
|  | Bi-factor | 28 | 92 | 1222.464 | 322 | <.001 | 0.962 | 0.955 | 0.018 |
|  |  |  |  |  |  |  |  |  |  |
| High SES  (N=3426) | Uni-factor | 28 | 64 | 1559.435 | 350 | <.001 | 0.85 | 0.837 | 0.032 |
|  | Correlated | 28 | 65 | 1363.328 | 349 | <.001 | 0.874 | 0.863 | 0.029 |
|  | Bi-factor | 28 | 92 | 611.677 | 322 | <.001 | 0.964 | 0.958 | 0.016 |
|  |  |  |  |  |  |  |  |  |  |
| Low SES  (N=4322) | Uni-factor | 28 | 64 | 1730.859 | 350 | <.001 | 0.879 | 0.87 | 0.03 |
|  | Correlated | 28 | 65 | 1598.133 | 349 | <.001 | 0.891 | 0.882 | 0.029 |
|  | Bi-factor | 28 | 92 | 755.941 | 322 | <.001 | 0.962 | 0.955 | 0.018 |

Note. Chi^2^ DF = Chi^2^ degree of freedom; CFI = Comparative fit index; TFI = Tucker-Lewis index; RMSEA = Root mean square error of approximation. Non-white populations were not included in subgroup analyses due to the small sample size and zero prevalence of some diseases.

**Table S19. The detailed item loading of the NCDS (population with disease) bi-factor model.**

| **Health condition** | D factor loading | Mental | Physical |
| --- | --- | --- | --- |
| ***Mental health condition*** |  |  |  |
| Depression | 0.500 (0.020) | 0.228 (0.025) |  |
| Anxiety | 0.442 (0.021) | 0.348 (0.024) |  |
| Phobia | 0.196 (0.024) | 0.383 (0.023) |  |
| Panic disorder | 0.220 (0.027) | 0.586 (0.029) |  |
| Irritability | 0.316 (0.019) | 0.086 (0.023) |  |
| Sleep problems | 0.130 (0.019) | 0.102 (0.022) |  |
| Forgetfulness | 0.632 (0.019) | 0.116 (0.027) |  |
| Eating disorders | 0.355 (0.081) | 0.030 (0.051) |  |
|  |  |  |  |
| ***Physical health condition*** |  |  |  |
| Fatigue | 0.444 (0.021) |  | 0.079 (0.023) |
| Migraine | 0.211 (0.027) |  | 0.241 (0.030) |
| Obesity | 0.096 (0.027) |  | 0.080 (0.032) |
| Heart problems | 0.210 (0.059) |  | 0.232 (0.073) |
| Hypertension | 0.028 (0.070) |  | 0.068 (0.092) |
| Diabetes | 0.163 (0.073) |  | -0.034 (0.075) |
| Eczema | 0.048 (0.033) |  | 0.216 (0.036) |
| Asthma | 0.113 (0.036) |  | 0.237 (0.041) |
| Hay fever | 0.025 (0.029) |  | 0.184 (0.032) |
| Ulcer | 0.116 (0.042) |  | 0.750 (0.035) |
| Gallstones | -0.011 (0.051) |  | 0.700 (0.051) |
| IBS | 0.200 (0.034) |  | 0.722 (0.027) |
| Ulcerative colitis / Crohn’s | -0.111 (0.079) |  | 0.981 (0.068) |
| Kidney / bladder stones | 0.219 (0.038) |  | 0.214 (0.049) |
| Back pain | 0.197 (0.025) |  | 0.204 (0.028) |
| Arthritis | 0.179 (0.030) |  | 0.261 (0.035) |
| Visual impairments | 0.081 (0.041) |  | 0.058 (0.049) |
| Hearing impairments | 0.228 (0.050) |  | 0.054 (0.066) |
| Tinnitus | 0.250 (0.029) |  | 0.185 (0.034) |
| Epilepsy | 0.415 (0.045) |  | 0.077 (0.064) |

Note. Grey means the results are not significant.

**Table S20. The detailed item loading of the NCDS (female) bi-factor model.**

| **Health condition** | D factor loading | Mental | Physical |
| --- | --- | --- | --- |
| ***Mental health condition*** |  |  |  |
| Depression | 0.611 (0.011) | 0.215 (0.017) |  |
| Anxiety | 0.547 (0.012) | 0.322 (0.017) |  |
| Phobia | 0.310 (0.015) | 0.391 (0.016) |  |
| Panic disorder | 0.244 (0.017) | 0.640 (0.023) |  |
| Irritability | 0.554 (0.012) | 0.037 (0.018) |  |
| Sleep problems | 0.546(0.014) | 0.055 (0.020) |  |
| Forgetfulness | 0.632 (0.010) | 0.132 (0.015) |  |
| Eating disorders | 0.284 (0.061) | -0.006 (0.053) |  |
|  |  |  |  |
| ***Physical health condition*** |  |  |  |
| Fatigue | 0.688 (0.014) |  | 0.025 (0.021) |
| Migraine | 0.227 (0.023) |  | 0.241 (0.034) |
| Obesity | 0.081 (0.023) |  | 0.122 (0.034) |
| Heart problems | 0.130 (0.053) |  | 0.201 (0.085) |
| Hypertension | 0.052 (0.054) |  | 0.161 (0.093) |
| Diabetes | 0.040 (0.061) |  | 0.153 (0.103) |
| Eczema | 0.087 (0.028) |  | 0.169 (0.041) |
| Asthma | 0.195(0.030) |  | 0.154 (0.047) |
| Hay fever | 0.036 (0.024) |  | 0.128 (0.035) |
| Ulcer | 0.166 (0.036) |  | 0.640 (0.052) |
| Gallstones | 0.117 (0.042) |  | 0.541 (0.067) |
| IBS | 0.215 (0.028) |  | 0.698 (0.038) |
| Ulcerative colitis / Crohn’s | -0.007 (0.066) |  | 0.901 (0.089) |
| Kidney / bladder stones | 0.221 (0.033) |  | 0.206 (0.057) |
| Back pain | 0.253 (0.021) |  | 0.225 (0.031) |
| Arthritis | 0.141 (0.027) |  | 0.310 (0.038) |
| Visual impairments | 0.053 (0.035) |  | -0.029 (0.053) |
| Hearing impairments | 0.148 (0.045) |  | 0.133 (0.063) |
| Tinnitus | 0.206 (0.025) |  | 0.200 (0.038) |
| Epilepsy | 0.259 (0.041) |  | 0.168 (0.070) |

Note. Grey means the results are not significant.

**Table S21. The detailed item loading of the NCDS (male) bi-factor model.**

| **Health condition** | D factor loading | Mental | Physical |
| --- | --- | --- | --- |
| ***Mental health condition*** |  |  |  |
| Depression | 0.583 (0.012) | 0.178 (0.021) |  |
| Anxiety | 0.558 (0.013) | 0.274 (0.023) |  |
| Phobia | 0.308 (0.017) | 0.305 (0.023) |  |
| Panic disorder | 0.220 (0.020) | 0.588 (0.038) |  |
| Irritability | 0.535 (0.013) | 0.077 (0.020) |  |
| Sleep problems | 0.512 (0.014) | 0.033 (0.019) |  |
| Forgetfulness | 0.592 (0.011) | 0.162 (0.019) |  |
| Eating disorders | 0.406 (0.073) | 0.012 (0.041) |  |
|  |  |  |  |
| ***Physical health condition*** |  |  |  |
| Fatigue | 0.652 (0.015) |  | 0.074 (0.020) |
| Migraine | 0.216 (0.023) |  | 0.256 (0.032) |
| Obesity | 0.080 (0.024) |  | 0.086 (0.033) |
| Heart problems | 0.208 (0.050) |  | 0.271 (0.081) |
| Hypertension | 0.148 (0.064) |  | 0.153 (0.131) |
| Diabetes | 0.187 (0.059) |  | -0.066 (0.081) |
| Eczema | 0.100 (0.027) |  | 0.239 (0.037) |
| Asthma | 0.122 (0.030) |  | 0.261 (0.043) |
| Hay fever | 0.043 (0.025) |  | 0.198 (0.033) |
| Ulcer | 0.190 (0.036) |  | 0.728 (0.039) |
| Gallstones | 0.104 (0.047) |  | 0.698 (0.060) |
| IBS | 0.227 (0.029) |  | 0.693 (0.031) |
| Ulcerative colitis / Crohn’s | 0.069 (0.064) |  | 0.936 (0.073) |
| Kidney / bladder stones | 0.194 (0.035) |  | 0.251 (0.051) |
| Back pain | 0.231 (0.022) |  | 0.209 (0.029) |
| Arthritis | 0.246 (0.025) |  | 0.251 (0.037) |
| Visual impairments | 0.100 (0.035) |  | 0.111 (0.052) |
| Hearing impairments | 0.081 (0.043) |  | 0.091 (0.074) |
| Tinnitus | 0.236 (0.025) |  | 0.204 (0.035) |
| Epilepsy | 0.252 (0.042) |  | 0.153 (0.077) |

Note. Grey means the results are not significant.

**Table S22. The detailed item loading of the NCDS (white population) bi-factor model.**

| **Health condition** | D factor loading | Mental | Physical |
| --- | --- | --- | --- |
| ***Mental health condition*** |  |  |  |
| Depression | 0.598 (0.009) | 0.202 (0.014) |  |
| Anxiety | 0.550 (0.009) | 0.311 (0.014) |  |
| Phobia | 0.306 (0.012) | 0.353 (0.014) |  |
| Panic disorder | 0.229 (0.013) | 0.606 (0.020) |  |
| Irritability | 0.543 (0.009) | 0.055 (0.014) |  |
| Sleep problems | 0.531 (0.010) | 0.047 (0.014) |  |
| Forgetfulness | 0.618 (0.007) | 0.135 (0.012) |  |
| Eating disorders | 0.320 (0.048) | 0.022 (0.034) |  |
|  |  |  |  |
| ***Physical health condition*** |  |  |  |
| Fatigue | 0.668 (0.011) |  | 0.054 (0.015) |
| Migraine | 0.221 (0.017) |  | 0.242 (0.024) |
| Obesity | 0.084 (0.017) |  | 0.104 (0.024) |
| Heart problems | 0.161 (0.038) |  | 0.222 (0.060) |
| Hypertension | 0.105 (0.044) |  | 0.141 (0.077) |
| Diabetes | 0.120 (0.043) |  | 0.000 (0.061) |
| Eczema | 0.093 (0.020) |  | 0.225 (0.028) |
| Asthma | 0.161 (0.022) |  | 0.229 (0.033) |
| Hay fever | 0.040 (0.018) |  | 0.177 (0.025) |
| Ulcer | 0.174(0.026) |  | 0.684 (0.034) |
| Gallstones | 0.116 (0.032) |  | 0.611 (0.048) |
| IBS | 0.219 (0.021) |  | 0.697 (0.025) |
| Ulcerative colitis / Crohn’s | 0.027 (0.048) |  | 0.906 (0.060) |
| Kidney / bladder stones | 0.210 (0.024) |  | 0.209 (0.040) |
| Back pain | 0.241 (0.015) |  | 0.218 (0.022) |
| Arthritis | 0.192 (0.019) |  | 0.271 (0.028) |
| Visual impairments | 0.081 (0.025) |  | 0.041 (0.038) |
| Hearing impairments | 0.118 (0.032) |  | 0.067 (0.049) |
| Tinnitus | 0.224 (0.018) |  | 0.196 (0.027) |
| Epilepsy | 0.260 (0.030) |  | 0120 (0.051) |

Note. Grey means the results are not significant.

**Table S23. The detailed item loading of the NCDS (high socio-economic status) bi-factor model.**

| **Health condition** | D factor loading | Mental | Physical |
| --- | --- | --- | --- |
| ***Mental health condition*** |  |  |  |
| Depression | 0.599 (0.014) | 0.202 (0.022) |  |
| Anxiety | 0.523 (0.016) | 0.362 (0.025) |  |
| Phobia | 0.266 (0.019) | 0.367 (0.020) |  |
| Panic disorder | 0.181 (0.021) | 0.499 (0.026) |  |
| Irritability | 0.530 (0.015) | 0.085 (0.024) |  |
| Sleep problems | 0.523 (0.017) | 0.067 (0.024) |  |
| Forgetfulness | 0.601 (0.012) | 0.143 (0.020) |  |
| Eating disorders | 0.311 (0.087) | 0.082 (0.086) |  |
|  |  |  |  |
| ***Physical health condition*** |  |  |  |
| Fatigue | 0.677 (0.017) |  | 0.055 (0.022) |
| Migraine | 0.185 (0.028) |  | 0.242 (0.038) |
| Obesity | 0.067 (0.028) |  | 0.093 (0.038) |
| Heart problems | 0.186 (0.028) |  | 0.165 (0.097) |
| Hypertension | -0112 (0.065) |  | 0.277 (0.124) |
| Diabetes | 0.035 (0.083) |  | 0.039 (0.123) |
| Eczema | 0.080 (0.032) |  | 0.233 (0.042) |
| Asthma | 0.128 (0.034) |  | 0.250 (0.050) |
| Hay fever | 0.047 (0.028) |  | 0.211 (0.038) |
| Ulcer | 0.164 (0.042) |  | 0.715 (0.047) |
| Gallstones | 0.113 (0.050) |  | 0.641 (0.075) |
| IBS | 0.238 (0.033) |  | 0.745 (0.032) |
| Ulcerative colitis / Crohn’s | 0.191 (0.067) |  | 0.977 (0.073) |
| Kidney / bladder stones | 0.150 (0.043) |  | 0.148 (0.060) |
| Back pain | 0.218 (0.025) |  | 0.213 (0.035) |
| Arthritis | 0.210 (0.030) |  | 0.238 (0.043) |
| Visual impairments | 0.081 (0.040) |  | 0.089(0.059) |
| Hearing impairments | 0.005 (0.053) |  | 0.047 (0.075) |
| Tinnitus | 0.188 (0.030) |  | 0.226 (0.042) |
| Epilepsy | 0.228 (0.046) |  | 0.169 (0.072) |

Note. Grey means the results are not significant.

**Table S24. The detailed item loading of the NCDS (low socio-economic status) bi-factor model.**

| **Health condition** | D factor loading | Mental | Physical |
| --- | --- | --- | --- |
| ***Mental health condition*** |  |  |  |
| Depression | 0.613 (0.012) | 0.221 (0.018) |  |
| Anxiety | 0.566 (0.012) | 0.268(0.018) |  |
| Phobia | 0.323 (0.015) | 0.371 (0.020) |  |
| Panic disorder | 0.236 (0.018) | 0.694 (0.032) |  |
| Irritability | 0.558 (0.012) | 0.045 (0.017) |  |
| Sleep problems | 0.526 (0.014) | 0.035 (0.019) |  |
| Forgetfulness | 0.630 (0.010) | 0.123 (0.015) |  |
| Eating disorders | 0.340 (0.066) | -0.086 (0.055) |  |
|  |  |  |  |
| ***Physical health condition*** |  |  |  |
| Fatigue | 0.665 (0.015) |  | 0.066 (0.023) |
| Migraine | 0.245 (0.024) |  | 0.223 (0.035) |
| Obesity | 0.095 (0.024) |  | 0.107 (0.036) |
| Heart problems | 0.119 (0.058) |  | 0.190 (0.087) |
| Hypertension | 0.199 (0.059) |  | 0.051 (0.133) |
| Diabetes | 0.126 (0.057) |  | 0.005 (0.082) |
| Eczema | 0.109 (0.029) |  | 0.214 (0.042) |
| Asthma | 0.147 (0.032) |  | 0.192 (0.049) |
| Hay fever | 0.014 (0.026) |  | 0.158 (0.036) |
| Ulcer | 0.171 (0.038) |  | 0.641 (0.055) |
| Gallstones | 0.093 (0.048) |  | 0.557 (0.070) |
| IBS | 0.219 (0.030) |  | 0.646 (0.041) |
| Ulcerative colitis / Crohn’s | -0.113(0.078) |  | 0.914 (0.100) |
| Kidney / bladder stones | 0.265 (0.033) |  | 0.278 (0.058) |
| Back pain | 0.253 (0.022) |  | 0.238 (0.032) |
| Arthritis | 0.188 (0.027) |  | 0.300 (0.041) |
| Visual impairments | 0.079 (0.037) |  | -0.040 (0.055) |
| Hearing impairments | 0.124 (0.044) |  | 0.088 (0.071) |
| Tinnitus | 0.213 (0.026) |  | 0.169 (0.039) |
| Epilepsy | 0.250 (0.042) |  | 0.123 (0.093) |

Note. Grey means the results are not significant.

**Table S25. Model fit of all ELSA subgroups.**

|  | **Model** | **Items** | **Free parameters** | **Chi^2^ Value** | **Chi^2^ DF** | **Chi^2^ p-value** | **CFI** | **TFI** | **RMSEA** |
| --- | --- | --- | --- | --- | --- | --- | --- | --- | --- |
| Diseased  (N=5950) | Uni-factor | 19 | 38 | 980.17 | 152 | <.001 | 0.847 | 0.828 | 0.03 |
|  | Correlated | 19 | 39 | 713.071 | 151 | <.001 | 0.896 | 0.882 | 0.025 |
|  | Bi-factor | 19 | 57 | 464.878 | 133 | <.001 | 0.939 | 0.921 | 0.02 |
|  |  |  |  |  |  |  |  |  |  |
| Female  (N=4199) | Uni-factor | 19 | 38 | 1099.914 | 152 | <.001 | 0.804 | 0.779 | 0.039 |
|  | Correlated | 19 | 39 | 598.303 | 151 | <.001 | 0.907 | 0.895 | 0.027 |
|  | Bi-factor | 19 | 57 | 278.458 | 133 | <.001 | 0.97 | 0.961 | 0.016 |
|  |  |  |  |  |  |  |  |  |  |
| Male  (N=3386) | Uni-factor | 18 | 36 | 1129.91 | 135 | <.001 | 0.681 | 0.638 | 0.047 |
|  | Correlated | 18 | 37 | 869.01 | 134 | <.001 | 0.764 | 0.731 | 0.04 |
|  | Bi-factor | 18 | 54 | 370.386 | 117 | <.001 | 0.919 | 0.894 | 0.025 |
|  |  |  |  |  |  |  |  |  |  |
| White  (N=7055) | Uni-factor | 19 | 38 | 1696.285 | 152 | <.001 | 0.778 | 0.75 | 0.038 |
|  | Correlated | 19 | 39 | 671.999 | 151 | <.001 | 0.925 | 0.915 | 0.022 |
|  | Bi-factor | 19 | 57 | 397.901 | 133 | <.001 | 0.962 | 0.951 | 0.017 |
|  |  |  |  |  |  |  |  |  |  |
| Non-White  (N=460) | Uni-factor | 17 | 34 | 379.718 | 119 | <.001 | 0.857 | 0.836 | 0.069 |
|  | Correlated | 17 | 35 | 366.245 | 118 | <.001 | 0.864 | 0.843 | 0.068 |
|  | Bi-factor | 17 | 51 | 151.055 | 102 | <.001 | 0.973 | 0.964 | 0.032 |
|  |  |  |  |  |  |  |  |  |  |
| Paid Job  (N=2565) | Uni-factor | 18 | 36 | 1421.571 | 135 | <.001 | 0.52 | 0.456 | 0.061 |
|  | Correlated | 18 | 37 | 1384.054 | 134 | <.001 | 0.534 | 0.467 | 0.06 |
|  | Bi-factor | 18 | 54 | 457.895 | 117 | <.001 | 0.873 | 0.834 | 0.034 |
|  |  |  |  |  |  |  |  |  |  |
| Not Job  (N=4990) | Uni-factor | 19 | 38 | 1184.443 | 152 | <.001 | 0.81 | 0.786 | 0.037 |
|  | Correlated | 19 | 39 | 568.725 | 151 | <.001 | 0.923 | 0.913 | 0.024 |
|  | Bi-factor | 19 | 57 | 354.17 | 133 | <.001 | 0.959 | 0.948 | 0.018 |

Note. Chi^2^ DF = Chi^2^ degree of freedom; CFI = Comparative fit index; TFI = Tucker-Lewis index; RMSEA = Root mean square error of approximation. Due to some groups got zero prevalence of some diseases, schizophrenia was not included in Male, Non-White, and Paid Job group and blood disorder was not included in Paid Job group.

**Table S26. The detailed item loading of the ELSA (population with disease) bi-factor model.**

| **Health condition** | D factor loading | Mental | Physical |
| --- | --- | --- | --- |
| ***Mental health condition*** |  |  |  |
| Depression | 0.21 (0.036) | 0.857 (0.019) |  |
| Anxiety | 0.175 (0.038) | 0.914 (0.019) |  |
| Emotional | 0.152 (0.051) | 0.813 (0.021) |  |
| Schizophrenia | 0.064 (0.098) | 0.436 (0.082) |  |
| Psychosis | 0.475 (0.094) | 0.481 (0.087) |  |
| Bipolar | 0.293 (0.082) | 0.430 (0.070) |  |
|  |  |  |  |
| ***Physical health condition*** |  |  |  |
| Stroke | 0.136 (0.083) |  | 0.522 (0.050) |
| Hypertension | -0.463 (0.079) |  | 0.535 (0.075) |
| Heart problems | 0.077 (0.064) |  | 0.420 (0.037) |
| Lung | 0.526 (0.060) |  | 0.312 (0.082) |
| Asthma | 0.353 (0.035) |  | 0.039 (0.055) |
| Arthritis | 0.165 (0.030) |  | 0.002 (0.036) |
| Osteoporosis | 0.322 (0.042) |  | 0.106 (0.056) |
| Blood disorder | 0.135 (0.074) |  | 0.073 (0.078) |
| Cancer | 0.116 (0.035) |  | -0.048 (0.038) |
| Parkinson's | 0.190 (0.061) |  | 0.032 (0.098) |
| Multiple Sclerosis | 0.353 (0.065) |  | -0.220 (0.129) |
| Dementia | 0.178 (0.053) |  | 0.285 (0.072) |
| Diabetes | -0.137 (0.059) |  | 0.356 (0.040) |

Note. Grey means the results are not significant.

**Table S27. The detailed item loading of the ELSA (female) bi-factor model.**

| **Health condition** | D factor loading | Mental | Physical |
| --- | --- | --- | --- |
| ***Mental health condition*** |  |  |  |
| Depression | 0.389 (0.045) | 0.814 (0.028) |  |
| Anxiety | 0.272 (0.049) | 0.887 (0.027) |  |
| Emotional | 0.255 (0.058) | 0.812 (0.030) |  |
| Schizophrenia | 0.838 (0.047) | 0.386 (0.049) |  |
| Psychosis | 0.587 (0.049) | 0.426 (0.109) |  |
| Bipolar | 0.343 (0.062) | 0.360 (0.085) |  |
|  |  |  |  |
| ***Physical health condition*** |  |  |  |
| Stroke | 0.215 (0.034) |  | 0.496 (0.044) |
| Hypertension | -0.013 (0.063) |  | 0.693 (0.050) |
| Heart problems | 0.243 (0.055) |  | 0.474 (0.041) |
| Lung | 0.623 (0.058) |  | 0.224 (0.064) |
| Asthma | 0.442 (0.046) |  | 0.126 (0.048) |
| Arthritis | 0.330 (0.045) |  | 0.338 (0.038) |
| Osteoporosis | 0.394 (0.0049) |  | 0.277 (0.047) |
| Blood disorder | 0.307 (0.081) |  | 0.106 (0.087) |
| Cancer | 0.212 (0.045) |  | 0.162 (0.040) |
| Parkinson's | 0.536 (0.052) |  | -0.069 (0.121) |
| Multiple Sclerosis | 0.454 (0.046) |  | -0.231 (0.117) |
| Dementia | 0.369 (0.045) |  | 0.217 (0.076) |
| Diabetes | 0.042 (0.060) |  | 0.468 (0.041) |

Note. Grey means the results are not significant.

**Table S28. The detailed item loading of the ELSA (male) bi-factor model.**

| **Health condition** | D factor loading | Mental | Physical |
| --- | --- | --- | --- |
| ***Mental health condition*** |  |  |  |
| Depression | 0.073 (0.040) | 0.906 (0.028) |  |
| Anxiety | 0.123 (0.040) | 0.944 (0.027) |  |
| Emotional | 0.231 (0.072) | 0.809 (0.031) |  |
| Psychosis | 0.660 (0.045) | 0.331 (0.122) |  |
| Bipolar | 0.637 (0.048) | 0.380 (0.107) |  |
|  |  |  |  |
| ***Physical health condition*** |  |  |  |
| Stroke | 0.216 (0.041) |  | 0.398 (0.050) |
| Hypertension | 0.503 (0.068) |  | 0.413 (0.058) |
| Heart problems | 0.215 (0.056) |  | 0.544 (0.040) |
| Lung | 0.096 (0.044) |  | 0.366 (0.045) |
| Asthma | -0.080 (0.044) |  | 0.308 (0.049) |
| Arthritis | 0.034 (0.065) |  | 0.482 (0.047) |
| Osteoporosis | 0.173 (0.047) |  | 0.407 (0.065) |
| Blood disorder | 0.351 (0.043) |  | 0.107 (0.077) |
| Cancer | -0.011 (0.046) |  | 0.258 (0.038) |
| Parkinson's | 0.407 (0.043) |  | -0.103 (0.109) |
| Multiple Sclerosis | 0.736 (0.040) |  | -0.468 (0.074) |
| Dementia | 0.361 (0.041) |  | 0.060 (0.071) |
| Diabetes | 0.523 (0.065) |  | 0.256 (0.063) |

Note. Grey means the results are not significant; Schizophrenia was not included because zero prevalence of it in the ELSA male group.

**Table S29. The detailed item loading of the ELSA (white population) bi-factor model.**

| **Health condition** | D factor loading | Mental | Physical |
| --- | --- | --- | --- |
| ***Mental health condition*** |  |  |  |
| Depression | 0.289 (0.035) | 0.841 (0.021) |  |
| Anxiety | 0.256 (0.037) | 0.902 (0.020) |  |
| Emotional | 0.246 (0.049) | 0.804 (0.023) |  |
| Schizophrenia | 0.722 (0.083) | 0.404 (0.081) |  |
| Psychosis | 0.458 (0.088) | 0.500 (0.093) |  |
| Bipolar | 0.372 (0.081) | 0.401 (0.072) |  |
|  |  |  |  |
| ***Physical health condition*** |  |  |  |
| Stroke | 0.264 (0.056) |  | 0.445 (0.042) |
| Hypertension | 0.096 (0.063) |  | 0.712 (0.041) |
| Heart problems | 0.266 (0.050) |  | 0.455 (0.037) |
| Lung | 0.615 (0.041) |  | 0.095 (0.062) |
| Asthma | 0.477 (0.034) |  | -0.005 (0.049) |
| Arthritis | 0.407 (0.033) |  | 0.232 (0.041) |
| Osteoporosis | 0.482 (0.039) |  | 0.114 (0.052) |
| Blood disorder | 0.215 (0.069) |  | 0.130 (0.069) |
| Cancer | 0.226 (0.034) |  | 0.108 (0.034) |
| Parkinson's | 0.244 (0.060) |  | 0.042 (0.080) |
| Multiple Sclerosis | 0.371 (0.061) |  | -0.324 (0.098) |
| Dementia | 0.223 (0.052) |  | 0.222 (0.064) |
| Diabetes | 0.108 (0.054) |  | 0.508 (0.035) |

Note. Grey means the results are not significant.

**Table S30. The detailed item loading of the ELSA (non-white population) bi-factor model.**

| **Health condition** | D factor loading | Mental | Physical |
| --- | --- | --- | --- |
| ***Mental health condition*** |  |  |  |
| Depression | 0.397 (0.058) | 0.896 (0.050) |  |
| Anxiety | 0.348 (0.055) | 0.913 (0.055) |  |
| Emotional | 0.558 (0.072) | 0.690 (0.096) |  |
| Psychosis | 0.853 (0.022) | 0.017 (0.083) |  |
| Bipolar | 0.865 (0.023) | 0.391 (0.096) |  |
|  |  |  |  |
| ***Physical health condition*** |  |  |  |
| Stroke | 0.569 (0.047) |  | 0.258 (0.134) |
| Hypertension | 0.464 (0.122) |  | 0.467 (0.089) |
| Heart problems | 0.304 (0.050) |  | 0.489 (0.082) |
| Lung | 0.508 (0.069) |  | -0.197 (0.124) |
| Asthma | 0.165 (0.052) |  | 0.210 (0.097) |
| Arthritis | 0.039 (0.047) |  | 0.751 (0.088) |
| Osteoporosis | 0.317 (0.052) |  | 0.563 (0.108) |
| Cancer | 0.260 (0.054) |  | 0.208 (0.101) |
| Parkinson's | 0.748 (0.040) |  | -0.285 (0.051) |
| Multiple Sclerosis | 0.860 (0.019) |  | -0.137 (0.034) |
| Dementia | 0.660 (0.060) |  | -0.174 (0.065) |
| Diabetes | 0.312 (0.109) |  | 0.296 (0.094) |

Note. Grey means the results are not significant; Schizophrenia and blood disorder were not included because zero prevalence of them in the ELSA non-white group.

**Table S31. The detailed item loading of the ELSA (with paid job) bi-factor model.**

| **Health condition** | D factor loading | Mental | Physical |
| --- | --- | --- | --- |
| ***Mental health condition*** |  |  |  |
| Depression | 0.169 (0.038) | 0.831 (0.033) |  |
| Anxiety | 0.101 (0.044) | 0.993 (0.035) |  |
| Emotional | 0.318 (0.049) | 0.804 (0.036) |  |
| Psychosis | 0.970 (0.085) | 0.242 (0.048) |  |
| Bipolar | 0.642 (0.039) | 0.178 (0.145) |  |
|  |  |  |  |
| ***Physical health condition*** |  |  |  |
| Stroke | 0.399 (0.038) |  | 0.098 (0.053) |
| Hypertension | 0.173 (0.014) |  | 0.443 (0.058) |
| Heart problems | 0.313 (0.025) |  | 0.211 (0.062) |
| Lung | 0.492 (0.028) |  | -0.327 (0.073) |
| Asthma | 0.292 (0.019) |  | -0.313 (0.048) |
| Arthritis | 0.215 (0.015) |  | -0.031 (0.048) |
| Osteoporosis | 0.404 (0.029) |  | -0.176 (0.046) |
| Blood disorder | 0.664 (0.042) |  | -0.062 (0.077) |
| Cancer | 0.331 (0.023) |  | -0.256 (0.058) |
| Parkinson's | 0.602 (0.042) |  | 0.373 (0.046) |
| Multiple Sclerosis | 0.581 (0.046) |  | 0.239 (0.038) |
| Dementia | 0.633 (0.038) |  | 0.467 (0.042) |
| Diabetes | 0.334 (0.022) |  | 0.613 (0.077) |

Note. Grey means the results are not significant; Schizophrenia was not included because zero prevalence of it in the ELSA non-white group.

**Table S32. The detailed item loading of the ELSA (without paid job) bi-factor model.**

| **Health condition** | D factor loading | Mental | Physical |
| --- | --- | --- | --- |
| ***Mental health condition*** |  |  |  |
| Depression | 0.325 (0.040) | 0.836 (0.024) |  |
| Anxiety | 0.322 (0.043) | 0.888 (0.025) |  |
| Emotional | 0.284 (0.057) | 0.787 (0.028) |  |
| Schizophrenia | 0.679 (0.106) | 0.394 (0.089) |  |
| Psychosis | 0.419 (0.096) | 0.456 (0.099) |  |
| Bipolar | 0.410 (0.093) | 0.473 (0.074) |  |
|  |  |  |  |
| ***Physical health condition*** |  |  |  |
| Stroke | 0.285 (0.057) |  | 0.389 (0.047) |
| Hypertension | 0.034 (0.068) |  | 0.745 (0.054) |
| Heart problems | 0.253 (0.051) |  | 0.430 (0.040) |
| Lung | 0.584 (0.046) |  | 0.127 (0.063) |
| Asthma | 0.480 (0.038) |  | 0.059 (0.052) |
| Arthritis | 0.385 (0.036) |  | 0.210 (0.042) |
| Osteoporosis | 0.476 (0.042) |  | 0.058 (0.055) |
| Blood disorder | 0.159 (0.078) |  | 0.052 (0.074) |
| Cancer | 0.183 (0.037) |  | 0.041 (0.035) |
| Parkinson's | 0.191 (0.064) |  | -0.060 (0.089) |
| Multiple Sclerosis | 0.357 (0.070) |  | -0.318 (0.123) |
| Dementia | 0.184 (0.059) |  | 0.152 (0.067) |
| Diabetes | 0.085 (0.057) |  | 0.487 (0.040) |

Note. Grey means the results are not significant.
